# A stepwise guide for pangenome development in crop plants: an alfalfa (*Medicago sativa*) case study

**DOI:** 10.1186/s12864-024-10931-w

**Published:** 2024-10-31

**Authors:** Harpreet Kaur, Laura M. Shannon, Deborah A. Samac

**Affiliations:** 1 Department of Horticultural Science, University of Minnesota, St. Paul, MN 55108 USA; 2 USDA-ARS, Plant Science Research Unit, St. Paul, MN 55108 USA

**Keywords:** Crop pangenome, Polyploids, Autotetraploid, Alfalfa, Graph-based pangenome

## Abstract

**Background:**

The concept of pangenomics and the importance of structural variants is gaining recognition within the plant genomics community. Due to advancements in sequencing and computational technology, it has become feasible to sequence the entire genome of numerous individuals of a single species at a reasonable cost. Pangenomes have been constructed for many major diploid crops, including rice, maize, soybean, sorghum, pearl millet, peas, sunflower, grapes, and mustards. However, pangenomes for polyploid species are relatively scarce and are available in only few crops including wheat, cotton, rapeseed, and potatoes.

**Main body:**

In this review, we explore the various methods used in crop pangenome development, discussing the challenges and implications of these techniques based on insights from published pangenome studies. We offer a systematic guide and discuss the tools available for constructing a pangenome and conducting downstream analyses. Alfalfa, a highly heterozygous, cross pollinated and autotetraploid forage crop species, is used as an example to discuss the concerns and challenges offered by polyploid crop species. We conducted a comparative analysis using linear and graph-based methods by constructing an alfalfa graph pangenome using three publicly available genome assemblies. To illustrate the intricacies captured by pangenome graphs for a complex crop genome, we used five different gene sequences and aligned them against the three graph-based pangenomes. The comparison of the three graph pangenome methods reveals notable variations in the genomic variation captured by each pipeline.

**Conclusion:**

Pangenome resources are proving invaluable by offering insights into core and dispensable genes, novel gene discovery, and genome-wide patterns of variation. Developing user-friendly online portals for linear pangenome visualization has made these resources accessible to the broader scientific and breeding community. However, challenges remain with graph-based pangenomes including compatibility with other tools, extraction of sequence for regions of interest, and visualization of genetic variation captured in pangenome graphs. These issues necessitate further refinement of tools and pipelines to effectively address the complexities of polyploid, highly heterozygous, and cross-pollinated species.

## Background

 Pangenomics is a relatively new field in comparative genomics. Unlike earlier genomic studies, which often focused on a single reference genome, pangenomics takes a more comprehensive approach by considering a broader range of the genetic variation present in each plant species and/or a genus and/or a clade which is crucial for understanding crop biology, evolution, and its adaptation to different environments. While historically single reference genomes were all that was practical, advances in high-throughput sequencing technologies and computational methods have made assembling whole genome sequences of numerous cultivars from a single species more accessible. With the advent of third generation sequencing technology (accurate long reads), it is now possible to accurately assemble multiple genomes of different cultivars at reasonable cost. The new sequencing data has revealed that different cultivars/accessions in a single species vary in their genome size and content (Table 1). In maize, genome size varies from 2.11 to 2.31 GB and repetitive content varies from 80.7 to 90.0% based on data from 17 cultivars [1–6]. This variation in genome size demonstrates that a single representative from a taxon cannot fully represent the entire genome of the taxa.

**Table 1:** Variation in genome size and repetitive elements in crops where multiple genotypes have been sequenced

Species	Cultivar/Accession	Genome Size (Gb)	Ploidy	Repetitive elements (%)	Reference
*Arabidopsis thaliana*	Col-00	0.13	2n = 2x = 10	23.87	Wang et al. [7]
	TAIR1010	0.12	2n = 2x = 10	21.00	Buisine et al. [8]
*Brassica napus*	Express 617	0.92	2n = 4x = 38	37.50	Lee et al. [9]
	Tapidor	0.63	2n = 4x = 38	35.15	Bayer et al. [10]
	Darmor-bzh	0.85	2n = 4x = 38	36.48–39.75	Bayer et al. [10, 11]
	ZS11	0.97	2n = 4x = 38	55.59	Sun et al. [11]
*Brassica oleracea* var. *capitata*	D134	0.57	2n = 2x = 18	55.25 TEs^a^	Lv et al. [12]
	TO1000	0.49	2n = 2x = 18	37.20 TEs	Parkin et al. [13]
	12 − 02	0.51	2n = 2x = 18	38.80 TEs	Liu et al. [14]
*Brassica rapa*	Chifu	0.42	2n = 2x = 20	53.78 TEs	Zhang et al. [15]
	PakChoi	0.41	2n = 2x = 20	63.30 TEs	Xu et al. [16]
	ECD04	0.35	2n = 2x = 20	42.71 TEs	Yang et al. [17]
	Z1	0.44	2n = 2x = 20	52.97 TEs	Istace et al. [18]
*Glycine max*	7 soybean cultivars	0.98-1.00	2n = 2x = 40	49.60-50.74	Chu et al. [19]
	Wm82	0.98	2n = 2x = 40	52.73	Valliyodan et al. [20, 21]
	JD17	0.96	2n = 2x = 40	54.03	Yi et al. [21]
	ZH13	1.02	2n = 2x = 40	56.25	Shen et al. [21, 22]
	Lee	1.01	2n = 2x = 40	48.15	Garg et al. [23]
*Glycine soja*	IGA1003	0.97	2n = 2x = 40	48.76	Chu et al. [19]
	W05	0.99	2n = 2x = 40	54.17	Xie et al. [24]
*Gossypium barbadense*	Pima90	2.21	2n = 4x = 52	62.10	Ma et al. [25]
	Hai7124	2.22	2n = 4x = 52	63.90 TEs	Hu et al. [26]
*Gossypium hirsutum*	TM-11	2.17	2n = 4x = 52	67.20	Li et al. [27]
	TM-11	2.29	2n = 4x = 52	62.20 TEs	Hu et al. [26]
	NDM8	2.29	2n = 4x = 52	61.85	Ma et al. [25]
*Hordeum vulgare*	Lasa Goumang	4.00	2n = 2x = 14	87.48	Zeng et al. [28]
	Morex	4.59	2n = 2x = 14	80.80 TEs	Mascher et al. [29]
	Golden Promise	4.13	2n = 2x = 14	72.88	Schreiber et al. [30]
*Lathyrus sativus*	Pusa-24	3.8	2n = 2x = 14	83.31	Rajarammohan et al. [31]
	LS007	6.2	2n = 2x = 14	70.78	Emmrich et al. [32]
*Medicago sativa*	ZhongmuNo.1	0.82	2n = 4x = 32	57.00	Shen et al. [33]
	ZhongmuNo.4	0.64 (2.56/4)	2n = 4x = 32	56.80	Long et al. [34]
	XinJiang DaYe	0.68 (2.73/4)	2n = 4x = 32	55.00	Chen et al. [35]
*Oryza sativa*	Basmati 334	0.39	2n = 2x = 24	44.40	Choi et al. [36]
	Dom Sufid	0.38	2n = 2x = 24	44.20	Choi et al. [36]
	R498	0.39	2n = 2x = 24	42.05	Du et al. [37]
	Nipponbare	0.37	2n = 2x = 24	40.03	Du et al. [37, 38]
*Pennisetum glaucum*	10 accessions	1.89-2.00	2n = 2x = 14	70.4–72.6	Yan et al. [39]
	Tift 23-D_2_B_1_-P_1_-P_5_	1.79	2n = 2x = 14	70.4–72.6	Varshney et al. [40]
*Solanum lycopersicum*	Heinz 1706 (SLT1.0)	0.80	2n = 2x = 24	65.66	Su et al. [41]
	Heinz 1706 (SL4.0)	0.78	2n = 2x = 24	71.77	Hosmani et al. [42]
	LA1673	0.80	2n = 2x = 24	66.20	Takei et al. [43]
*Solanum tuberosum*	Russian cultivars	0.65–1.30	2n = 4x = 48	59.72–62.65	Karetnikov et al. [44]
	ADG1	0.84	2n = 4x = 48	60.20	Kyriakidou et al. [45]
	DM1-3 (doubled monoploid)	0.73	2n = 2x = 24	62.20	Potato Genome Sequencing Consortium [46]
	Otava	3.1	2n = 4x = 48	66.0	Sun et al. [47]
	Qingshu No. 9	2.67	2n = 4x = 48	63.3	Wang et al. [48]
	C88	3.1	2n = 4x = 48	-	Bao et al. [49]
	Solyntus	0.72	2n = 4x = 48	-	Van Lieshout et al. [50]
*Sorghum bicolor*	V9	0.79^b^	2n = 2x = 20	55.49	Kuo et al. [51]
	WLWL^a^	0.80^b^	2n = 2x = 20	56.83	Kuo et al. [51]
	YLYL^a^	0.79^b^	2n = 2x = 20	54.68	Kuo et al. [51]
*Sorghum halepense*	TT	3.16^b^	2n = 4x = 40	54.68	Kuo et al. [51]
	US	3.10^b^	2n = 4x = 40	56.24	Kuo et al. [51]
*Triticum aestivum*	Chinese Spring	14.50	2n = 6x = 42	85.00	International Wheat Genome Sequencing Consortium, 2018 [52]
	10 reference-quality assemblies	14.30–14.80	2n = 6x = 42	80.80–81.40	Walkowiak et al. [53]
*Vicia sativa*	Studenica	1.65	2n = 2x = 12	83.92	Xi et al. [54]
	KSR5	1.54	2n = 2x = 14	51.9	Shirasawa et al. [55]
*Vigna radiata*	Vrad_JL7	0.47	2n = 2x = 22	53.45	Liu et al. [56]
	VC1973A	0.47	2n = 2x = 22	52.79	Ha et al. [56, 57]
*Zea mays*	A188	2.21	2n = 2x = 20	80.70	Ge et al. [5]
	B73	2.18	2n = 2x = 20	85.27	Hufford et al. [58]
	Mo17	2.18	2n = 2x = 20	88.37	Chen et al. [59]
	SK	2.16	2n = 2x = 20	90.00	Yang et al. [3]
	K0326Y	2.16	2n = 2x = 20	83.32	Li et al. [4]
	12 Founder Inbred Lines	2.17–2.31	2n = 2x = 20	83.94–85.56	Wang et al. [6]
	25 NAM Founders	2.12–2.30	2n = 2x = 20	85.21–85.78	Hufford et al. [58]

^a^*TEs* Transposable Elements

Pangenomes reveal how different individuals within a species vary at the genomic scale, which cannot be represented fully in a single reference genome. Even a comparison of fragmented assemblies generated using *de novo* approaches offers significant insight into structural variations (SVs) within coding sequences of genomes when compared to mapping reads to a single reference genome [60]. However, whole genome *de novo* assembly, which involves reconstructing the complete genome sequence of an organism without the use of a reference genome, remains a complex and challenging task. The presence of extensive repetitive sequence and high heterozygosity, especially in polyploid species, contribute to the difficulty of this process and results in highly fragmented assemblies with unknown completeness. In such situations, methods including reference genome-based resequencing, pan-transcriptomics [61] and metagenomics [62] can help to accurately assemble complete genomes and detect novel sequences. Techniques such as optical mapping, high throughput chromosome conformation capture (Hi-C), and genetic maps can resolve multiple haplotypes [47, 50], although, accurately assembling repetitive regions is still a challenge.

This review provides a summary of available methods and challenges encountered for developing and annotating a crop pangenome. We also present a case study for alfalfa (*Medicago sativa* L.), highlighting different methods for exploring the extent of variation in a pangenome of a highly heterozygous cross pollinated autopolyploid species.

## Main body

### What is a pangenome?

There are many different definitions of pangenome that exist within the scientific community. The word pan is derived from a Greek word ‘παν’, meaning whole. The first pangenome was developed by Tettelin et al. [63], who compared eight strains of the bacterium *Streptococcus agalactiae*. Since then, a pangenome approach has been applied to many higher organisms such as plants [64] and humans [65]. The concept of constructing a pangenome in plants was initially suggested by Morgante et al. [64] in 2007. The first report of a plant pangenome was published in maize in 2014 [61]. The first pangenome in a polyploid plant species was published in hexaploid bread wheat (*Triticum aestivum*) by Montenegro et al. [66] in 2017.

According to the most used nomenclature, a pangenome represents the entire set of genes within a species, consisting of a ‘core’ genome which comprises genes shared among all individuals (100%) of the species and the ‘dispensable’, ‘accessory’ or ‘variable’ portion which is present in only a few individuals (1–99%). Many researchers [56, 67–69] divide the dispensable part of the genome into soft core, shell, and cloud genes which are present in 95–98%, 5–94% and 1–5% of individuals, respectively. Similar classification (shell and cloud) of dispensable genes has been used by Bozan et al. [70] and Hoopes et al. [71] in the potato pangenome. Genes which are present in only one individual are termed ‘private genes’ [39] or ‘unique genes’ [68]. The cloud and private genes present in genomes could be artifacts and should be confirmed with RNA-seq data and by mapping raw reads back to the assembled genome. With RNA-seq analysis, only 22% of the total cloud genes present in 32 individuals identified in the pangenome of *Brachypodium distachyon* were expressed, suggesting that up to 78% may be artifacts of the analysis [67].

Pangenomics is a method for representing genetic diversity within a taxon defined at any level, either for the genome as a whole or a relevant subset of genes. A pangenome may represent global genetic diversity from multiple related species as shown in *Vitis* [72] and *Solanum* [70] pangenomes. At a smaller scale, a full repertoire of a class of genes such as disease resistance genes in a pangenome can be developed by identifying the genes containing the NB-ARC domain using NLR-parser [73] and InterProScan 5.0 [74]. This gene family specific pangenome analysis represents the core genes, domain diversity, new domain architectures, presence-absence polymorphisms, and novel genes in the family as demonstrated in *M. truncatula* [60] and cotton [75]. Similarly, other important gene families related to particular biotic or abiotic stress or root nodule specific genes (NCRs) can also be investigated by creating a pan-ome for that specific gene family, e.g. pan-NLRome [76].

In essence, the pangenome represents genetic diversity in a selected group of organisms where genomes of these organisms are combined to identify the minor to major genomic variations between the genomes such as single nucleotide polymorphism (SNPs), small insertions and deletions (INDELs), and major structural variations (SVs). The SVs including presence or absence variation (PAVs), copy number variation (CNVs), and other large chromosomal rearrangements like inversions and translocations are an important form of variation in plants [20, 66]. Most pangenome development tools have been initially designed for prokaryotic and mammalian genomes, with limited to no focus on plant genomes. Plant genomes have experienced much more dynamic evolutionary changes compared to animal genomes [77], largely due to frequent polyploidy events [78, 79], extensive chromosomal rearrangements [80], and a higher rate of structural variations such as duplications, deletions, and mutations [81]. This suggests that many available pangenome development tools are unable to accurately capture all the genomic variation within a single plant species.

The number of genotypes needed for a pangenome depends on the genetic diversity in the taxa the pangenome is meant to represent. Pangenomes can be understood as open or closed. In an open pangenome the number of gene families will continue to increase significantly with the addition of new genomes to the analysis, while in a closed pangenome additional genotypes will not meaningfully increase the number of gene families. Tettelin et al. [63], proposed the alpha value of Heaps’ law as a threshold to consider a pangenome either open or closed. Heaps’ Law, originally proposed by Gustav Herdan [82] and Harold Stanley Heaps [83] in the field of linguistics [82, 83], describes the relationship between the size of a vocabulary (the number of unique words) in a document and the length of that document. It states that the total number of the unique words ($\:n$) to be identified in a document is a function of length of the document ($\:N$) which according to the power-law function can be expressed as:

$$\:n=k{N}^{\alpha\:}$$

where:

$\:n$ is the total number of unique words in a document of length $\:N$.

$\:k$ is a constant representing the rate of vocabulary growth.

$\:\alpha\:$ is another constant representing the rate of increase in the vocabulary size as the document length increases.

In the context of genomics and bioinformatics, Heaps’ Law has been adapted to describe the growth of a pangenome. In this adaptation, the length of the document corresponds to the number of individuals (or genomes) sequenced, and the size of the vocabulary corresponds to the total number of unique genes or genetic elements identified across those individuals. Therefore, Heaps’ Law for a pangenome can be expressed with the same equation where $\:n$ is the total number of unique genes found for a given number of sequenced individuals $\:N$, $\:k$ and $\:\alpha\:$ are parameters similar to those in the original Heaps’ Law, representing the rate of growth of the pangenome and the rate of increase in the number of new genes with each additional individual sequenced. Heaps’ Law suggests that as more genomes are added to the pangenome, the number of unique genomic elements will grow, but at a diminishing rate. In other words, each additional genome contributes fewer new unique elements compared to the genomes already present in the pangenome. This reflects the idea that as more genomes are sequenced, the discovery of entirely new genetic elements becomes less frequent, as many common elements are already represented in the pangenome.

An alpha value < 1 is representative of an open pangenome and an alpha > 1 is representative of a closed pangenome. In a closed pangenome, the curve representing the increase in the size of pangenome reaches an asymptote when the number of newly added genes are modeled against the number of genomes sequenced. A closed pangenome has been developed in rice [84], sorghum [85], tomato [69], soybean [86], maize [61], peppers [87], sunflower [88], pea [68], chickpea [89], and Chinese mung bean [56]. The number of individuals used for the construction of these closed pangenomes ranged from 118 to 3,366 (Table 2). Many different types of cultivars, landraces and wild species were included in these analyses to maximize diversity. In one of the allotetraploid self-pollinated species, rapeseed, a closed pangenome was constructed using only 53 individuals [90]. Notably, pangenomes in other major polyploid crops such as allohexaploid wheat [66], allotetraploid cotton [75], and autotetraploid potato [71] have yet to reach a plateau. This may be due to the smaller number of genotypes (8–18) used in these studies compared to those in closed pangenomes, combined with relaxed selection in polyploids [91] that results in higher diversity in polyploids [92, 93] in particular structural diversity [94] and subgenome fractionation as part of the rediploidization process [95, 96].

**Table 2:** Gene-based pangenome studies in crops with varying number of genotypes, sequencing methods, and dispensable genes

Species	No. of accessions	Sequencing Method	Dispensable genes (%)	Reference
*Arabidopsis thaliana*	19	Illumina HiSeq	30.0	Contreras-Moreira et al. [97]
*Brachypodium distachyon*	54	Illumina HiSeq	27.0	Gordon et al. [67]
*Brassica napus*	53	Illumina HiSeq	38.0	Hurgobin et al. [90]
		Illumina Mate Pair (2 accessions)		
*Brassica rapa*	3	Illumina HiSeq	13.0	Lin et al. [98]
*Cajanus cajan*	89	Illumina HiSeq	14.0	Zhao et al. [99]
*Capsicum annuum*	12	Illumina HiSeq	53.8	Lee et al. [100]
		PacBio HiFi		
		Hi-C		
		BioNano Optical mapping		
		Nanopore for 11 cultivars		
*Capsicum* ssp.	383	Illumina HiSeq	44.3	Ou et al. [87]
*Cicer* ssp. (wild and cultivated)	3366	Illumina HiSeq	1,582^a^ novel genes	Varshney et al. [89]
*Cucumis sativus*	11	Illumina HiSeq	20.0	Li et al. [101]
		PacBio		
		Hi-C		
*Glycine max*	204	Illumina HiSeq	90.6	Torkamaneh et al. [86]
*G. soja*,* G. max*	27	Illumina HiSeq	49.9	Liu et al. [102]
		PacBio HiFi		
		Hi-C		
		BioNano Optical mapping		
*Glycine soja*	7	Illumina HiSeq	51.4	Li et al. [103]
*Gossypium* sp.	8	Illumina HiSeq	31.9	Peng et al. [75]
		PacBio		
		Hi-C		
*Helianthus annuus*	287	Illumina HiSeq	27.3	Hubner et al. [88]
*Medicago truncatula*	15	Illumina HiSeq	67.0	Zhou et al. [60]
*Oryza sativa*	3	Illumina HiSeq	8.0	Schatz et al. [104]
	239	Illumina HiSeq	1,131^a^ novel genes	Liu et al. [105]
	3010	Illumina HiSeq	46.5	Wang et al. [84]
		PacBio for reference genome		
	251	Illumina HiSeq	57.4	Shang et al. [106]
		Nanopore		
		Hi-C		
	3010	Illumina HiSeq	12,465^a^ novel genes	Hu et al. [107]
		PacBio for reference genome		
*Pennisetum glaucum*	11	Illumina HiSeq	39.7–49.9	Yan et al. [39]
		PacBio HiFi		
		Hi-C		
		BioNano Optical mapping		
*Pisum sativum*	118	Illumina HiSeq	64.8	Yang et al. [68]
*Raphanus* sp.	11	Illumina HiSeq	83.7	Zhang et al. [108]
		PacBio HiFi		
		Hi-C		
		BioNano Optical mapping		
*Sesamum indicum*	5	Illumina HiSeq	41.8	Yu et al. [109]
*Solanum lycopersicum*	725	Illumina HiSeq	25.8	Gao et al. [69]
	100	Illumina HiSeq		
		Oxford Nanopore		
*Solanum tuberosum*	15	Illumina HiSeq	33.9	Hoopes et al. [71]
*Sorghum bicolor*	354	Illumina HiSeq	53.0	Ruperao et al. [85]
	16	Illumina HiSeq	64.0	Tao et al. [110]
		PacBio		
		Hi-C		
*Triticum aestivum*	18	Illumina HiSeq	36.0	Montenegro et al. [66]
*Vigna radiata*	217	Illumina HiSeq	16.9	Liu et al. [56]
*Zea mays*	503	Illumina HiSeq	61.0	Hirsch et al. [61]
	14	PacBio	34.5	Wang et al. [6]
		BioNano Optical Maps		
	26	PacBio	72.9	Hufford et al. [58]
		BioNano Optical Maps		
*Zea* sp.	721	Illumina HiSeq	44.3	Gui et al. [111]

### Why a pangenome?

The main goal of developing a pangenome is to capture as much genetic diversity as possible. The importance of pangenomes is illustrated by the number of novel genes and dispensable genome fractions identified in various crops (Table 2) which varies from 8% among three *Oryza sativa* individuals to 83.7% among 11 *Raphanus* sp. individuals. The number of genes in a pangenome varies with the number of genotypes being compared, genomic diversity captured by selected individuals, the quality of genome assembly and its annotation, as well as the technology and tools used to sequence the genome and classify the core and dispensable fraction. As discussed by Golicz et al. [112], when a small number of related, non-diverse genotypes are selected, the pangenome significantly underestimates the sequence diversity of the taxon. Whereas, selecting the highly diverse, large dataset provides more comprehensive and realistic view of pangenome.

Since many genes/genomic fractions are absent in a single genome, a pangenome will decrease the single genome reference bias in plant breeding and gene editing experiments. As discussed by Della Coletta et al. [113], if variants associated with a trait are not present in a reference genome, then linkage based quantitative trait loci (QTL) mapping or genome wide association studies (GWAS) will not detect those variants, and true deletions will be assigned imputed genotype values. This bias becomes crucial in highly heterozygous, polyploid, and outcrossing species in which assembling a complete genome is a challenge, and genome assemblies are often incomplete or highly fragmented. The same is true for discovery of SVs when these are identified with discordant read-pair, split-reads, and read-depth approaches using a single reference genome. A detailed review on the importance of SVs in plant breeding has been done by Yuan et al. [114]. They discussed that SVs influence numerous agronomically important genes involved in key traits such as seed development, flowering time, quality, stress response, and chemical responses across various field and horticultural crops.

### Pangenome development methods

**Fig. 1:**
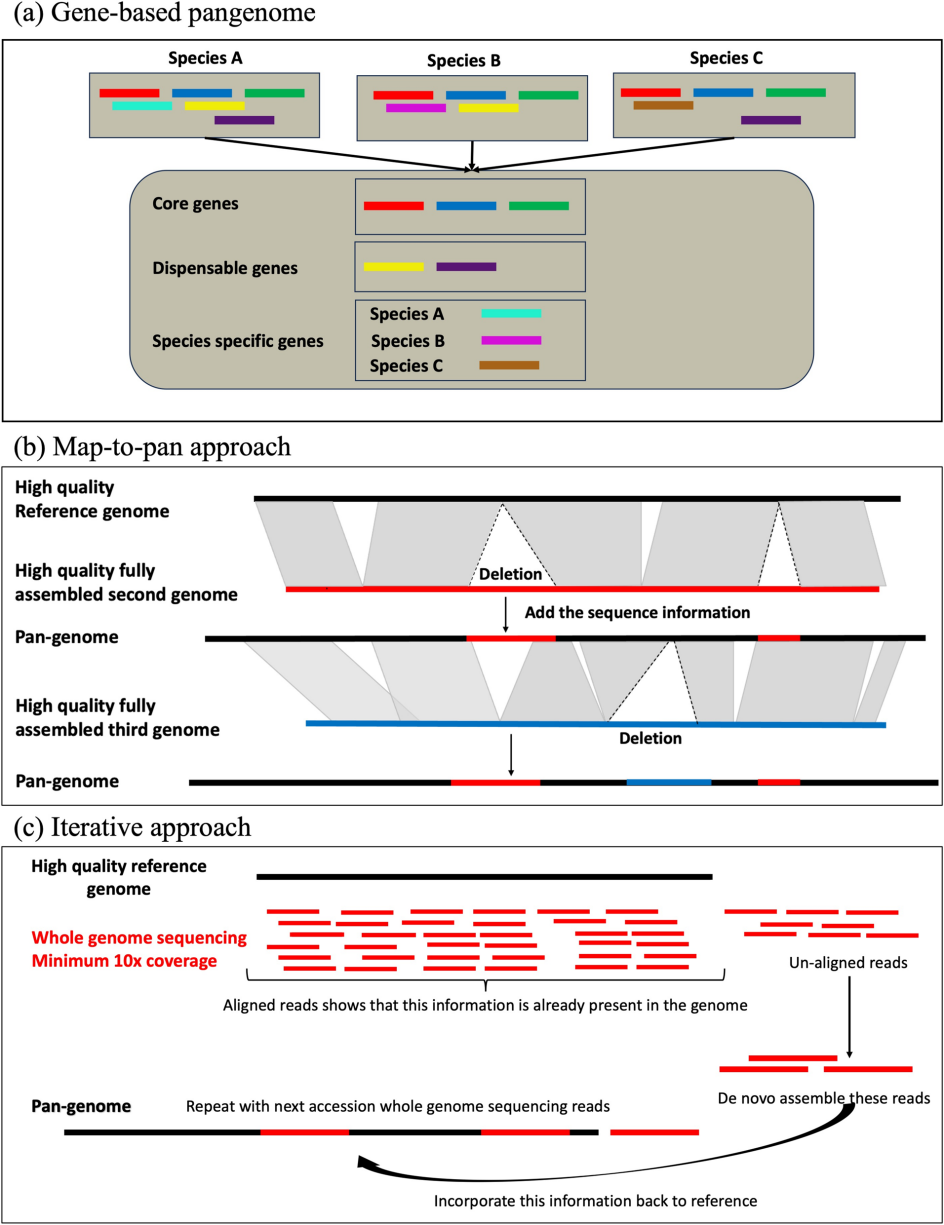
The pangenome development methods. Different colors represent different genes in Fig. **a**) and assemblies, in Fig. **b**), and **c**). Grey areas represent colinear segments between assemblies. **a** Gene-based pangenome approach, **b** Map-to-pan approach, **c** Iterative mapping approach

**Fig. 2:**
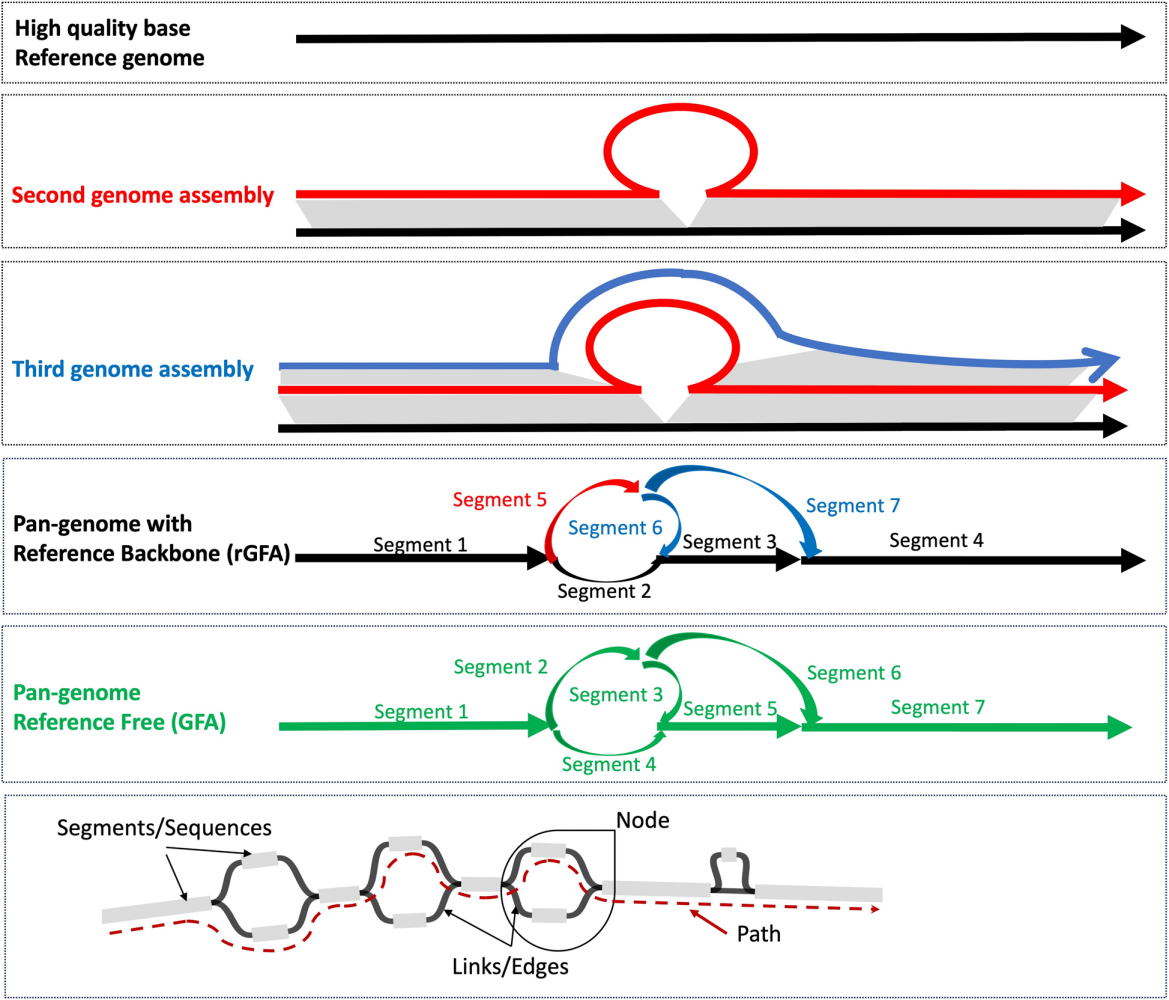
Graph-based pangenome development approach. In the reference Graphical Fragment Assembly (rGFA) format, used by Minigraph and Minigraph-Cactus pipelines, origin of the segment can be traced back to its linear genome used to build the graph and each segment/sequence is associated with only one origin. In GFA (v. 1.1 and 1.0) format, used by PanGenome Graph Building (PGGB) and k-mer based approaches, tracing the origin back to its linear genome is difficult. Segments in a pangenome graph are DNA sequences. Links/Edges connects the segments/sequences to each other and represent overlapping sequences between two segments. Links/Edges could be bidirected and describes the possible ways of walking through the nodes. Nodes are group of segments connected with edges through which multiple paths are possible

**Fig. 3:**
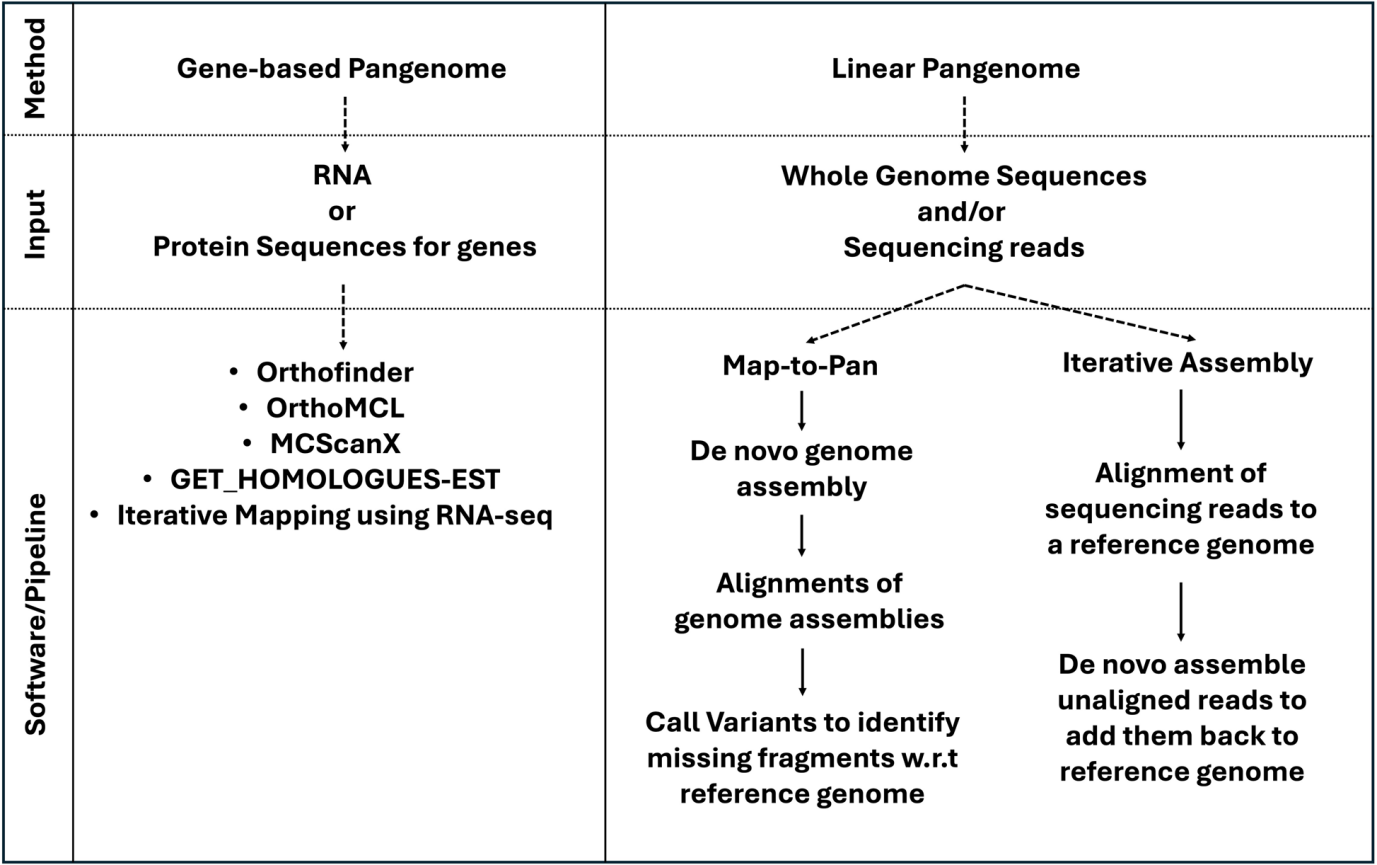
Flowcharts explaining gene-based and linear pangenome development pipelines or software. Different software used in the linear pangenome are mentioned in Table 3

**Fig. 4:**
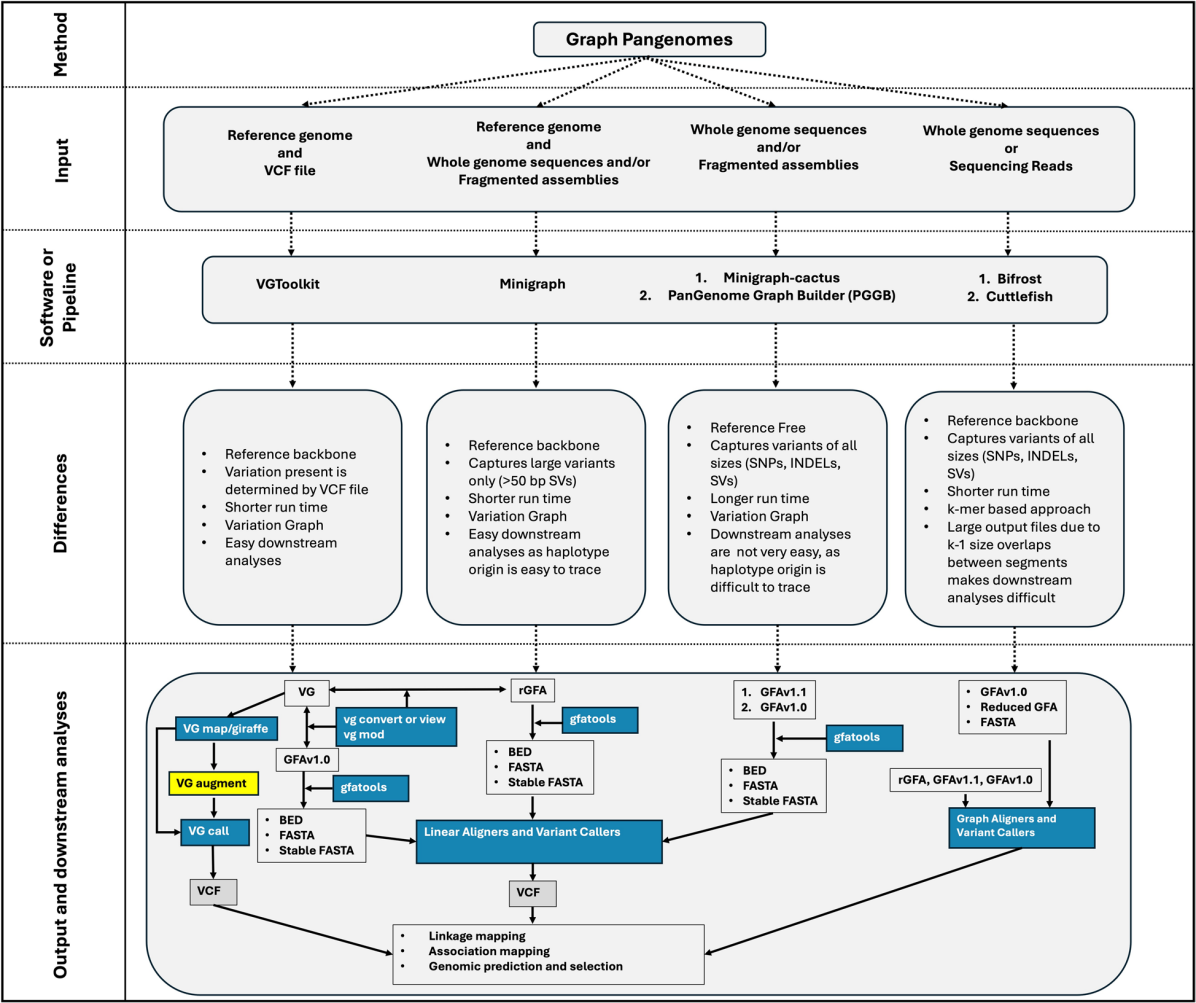
Flowchart explaining the five different pangenome graph building pipelines, their comparisons, and the downstream analyses. In output and downstream analyses section, blue boxes are the software tools and other boxes are outputs of these tools. VG augment function, highlighted yellow box, only works with VG output which should be generated using a reference genome and VCF file in VGToolkit. Double sided arrow means that the function works both ways i.e., VG format can be converted into rGFA and vice-versa. Other linear and graph alignment and variant calling tools are mentioned in Table 3

Multiple approaches have been proposed to develop pangenomes. The steps and tools involved in these approaches are summarized in Figs. 1, 2, 3 and 4; Table 3. These methods can be broadly classified into two groups: annotated gene/transcript based and whole genome sequence-based approaches.

**Table 3:** Steps and tools used in pangenome development

**Steps**	**Usage**	**Input**	Tools^*^	**Reference/ Gtihub Weblink**
Selection of genotypes	Phylogenetic analysis using clustering tools	Multi-sequence alignments	RAxML	Stamatakis [115]
			PhyML	Guindon et al. [116]
			FastTree	Price et al. [117]
Genome assembly	*De Novo* assemblers	Long reads (PacBio, ONT, Illumina Nextera)	HiFiasm	Cheng et al. [118], Cheng et al. [119]
			Canu	Koren et al. [120]
			SPAdes	Bankevich et al. [121]
			MECAT	Xiao et al. [122]
			Miniasm	Li [123]
			Wtdbg2	Ruan and Li [124]
			Raven	Vaser and Sikic [125]
		Polish long read assembly	Quiver	Chin et al. [126]
			CONSENT	Morisse et al. [127]
		Error-correction in long reads using short reads	Pilon	Walker et al. [128]
		Short reads (Illumina Mate-pair or HiSeq)	Velvet	Zerbino and Birney [129]
			SOAPdenovo2	Luo et al. [130]
			ABySS	Simpson et al. [131], Jackman et al. [132]
			Ray	Boisvert et al. [133]
			MEGAHIT	Li et al. [134]
		Mix short reads from multiple samples with uneven sequencing depth	IDBA-UD	Peng et al. [135]
		Short and long reads	ALLPATHS-LG	Gnerre et al. [136]
**Steps**	**Usage**	**Input**	Tools^*^	**Weblink**
			MaSuRCA	Zimin et al. [137]
	Order and scaffolding	Hi-C	3D-DNA	Dudchenko et al. [138]
			ALLHiC	Zhang et al. [139]
			LACHESIS	Zhang et al. [139]
			Juicer	Durand et al. [140]
		Bionano Optical Map	Kermit	Github Weblink [141]
			OpticalKermit	Github Weblink [142]
			Novo&Stitch	Github Weblink [143]
			OMGS	Github Weblink [144]
		Genetic Linkage Map	Kermit	Github Weblink [141]
Structural genome annotations	Evidence-based	Assembled genome	MAKER	Holt et al. [145]
		RNA sequencing data	BRAKER	Bruna et al. [146]
	ab initio	Assembled genome	Augustus	Stanke et al. [147]
			GeneID	Github Weblink [148]
			GlimmerHMM	Majoros et al. [149]
			SNAP	Korf [150]
Functional genome annotations	Homology-based	Gene sequences	InterproScan	Github Weblink [151]
			BLAST	Mount [152]
Genome alignments	Pairwise alignment	Short reads (< 1 Mbp) such as Illumina	BWA-mem	Li and Durbin [153]
			Bowtie2	Langmead and Salzberg [154]
			HISAT2	Kim et al. [155]
		Long reads (> 1 Mbp) such as PacBio, ONT	Minimap2	Li [156]
			NGMLR	Sedlazeck et al. [157]
**Steps**	**Usage**	**Input**	Tools^*^	**Weblink**
			BLASR	Github Weblink [158]
	Pairwise and single copy	Whole genome	MUMmer	Kurtz et al. [159]
			AnchorWave	Song et al. [160]
	Multiple sequence alignment	Long reads or whole genome	Progressive Cactus	Armstrong et al. [161]
			Mauve	Darling et al. [162]
			LASTZ	Github Weblink [163]
		Draft whole genome sequences	Mugsy	Angiuoli and Salzberg [164]
Detect variants	Structural Variants (> 50 bp)	Whole genome pairwise alignments	SyRI	Goel et al. [165]
			SVMU	Chakraborty et al. [166]
			Assemblytics (includes SNPs)	Nattestad and Schatz [167]
		Long reads/contigs alignment to a reference genome	Smartie-SV	Kronenberg et al. [168]
			Sniffles	Sedlazeck et al. [157], Smolka et al. [169]
			PBHoney	English et al. [170]
			NanoSV	Cretu Stancu et al. [171]
		Short reads alignments to a reference genome	Breakdancer	Fan et al. [172]
			Lumpy	Layer et al. [173]
			Delly	Rausch et al. [174]
	SNPs and small SVs (< 50 bp)	Multiple samples short read alignments to a reference genome	GATK	McKenna et al. [175]
			Freebayes	Garrison and Marth [176]
**Steps**	**Usage**	**Input**	Tools^*^	**Weblink**
			NGSEP	Perea et al. [177], Tello et al. [178]
	Genes presence-absence	Short reads alignment to reference genome	scanPAV	Giordano et al. [179]
			SGSGeneloss-based method	Tay Fernandez et al. [180]
			ppsPCP	Tahir Ul Qamar et al. [181]
Merge variants	All types	Multiple VCFs files	Bcftools merge	Danecek et al. [182]
			SURVIVOR	Github Weblink [183]
			PanPop	Zheng et al. [184]
		Multiple bed-like files	Bedtools	Quinlan and Hall [185]
			JASMINE	Github Weblink [186]
Pangenome development	Gene-based	Protein sequences (Clustering using complete gene models)	Orthofinder	Emms and Kelly [187]
			OrthoMCL	Li et al. [188]
		Protein Sequences (Clustering using fragmented gene models)	GET_HOMOLOGUES-EST	Contreras-Moreira et al. [97]
		Gene coding sequences and annotations (Synteny analyses)	MCScanX	Wang et al. [189]
	Linear-sequence based	Assemble unaligned short reads	*De Novo* Assemblers	
		Remove redundant sequences	CD-HIT	Fu et al. [190]
			BLASTn	Mount [152]
			PSVCP	Wang et al. [191]
	Graph-based	Reference genome and VCF	VG construct	Garrison et al. [192], Hickey et al. [193]
**Steps**	**Usage**	**Input**	Tools^*^	**Weblink**
		Multiple whole genomes in fasta format	Minigraph	Li et al. [194]
		Haplotype resolved multiple genomes in fasta format	Minigraph-Cactus	Hickey et al. [195]
			PGGB	Garrison et al. [196]
		Whole genome sequences or sequencing reads	Cuttlefish	Khan et al. [197]
			Bifrost	Holley and Melsted [198]
Mapping against pangenome as reference	Linear- sequence based alignments	Short reads (< 1 Mbp) such as Illumina	BWA-mem	Li and Durbin [153]
			Bowtie2	Langmead and Salzberg [154]
			HISAT2	Kim et al. [155]
		Long reads (> 1 Mbp) such as PacBio, ONT	Minimap2	Li [156]
			NGMLR	Sedlazeck et al. [157]
			BLASR	Github Weblink [158]
	Graph-based alignments	Convert GFA or rGFA to linear fasta	gfatools	https://github.com/lh3/gfatools
		Graph-based short reads mapping	VG giraffe	Siren et al. [199]
		Graph-based long reads mapping	GraphMap	Sovic et al. [200]
			GraphAligner	Rautiainen et al. [201]
Pangenome based variant calling	SNPs, INDELs and SVs	Linear alignments	GATK	McKenna et al. [175]
			Minigraph sequence-to-graph mapping	Li et al. [194]
			Freebayes	Garrison and Marth [176]
**Steps**	**Usage**	**Input**	Tools^*^	**Weblink**
			NGSEP	Perea et al. [177], Tello et al. [178]
			Any haplotype caller	
		Graph-based alignments (VG/GFA format)	VG Toolkit (pack, snarls, call)	Hickey et al. [193], Paten et al. [202]
		Short reads and reference graph (GFA format)	Paragraph	Chen et al. [203]
			GraphTyper / GraphTyper2	Eggertsson et al. [204, 205]
			PanGenie	Ebler et al. [206]
			BayesTyper	Sibbesen et al. [207]
Pangenome annotations	Linear Pangenome	FASTA sequence files	Any annotation tools mentioned above	
	Graph Pangenome	GFA file	ggCaller	Horsfield et al. [208]
			PanTools	Sheikhizadeh et al. [209], Anari et al. [210]
		VG file	VG rna, mpmap	Garrison et al. [192]
			RPVG	Sibbesen et al. [211]
Pangenome visualization	Graphical User Interface	GFA file	BANDAGE	Wick et al. [212]
			gfaviz	Gonnella et al. [213]
			PANACHE	Durant et al. [214]
	Command line interface	VG file	VG viz.	Garrison et al. [192]
			Sequence Tube Map	Github Weblink [215]
		ODGI file	ODGI	Guarracino et al. [216]

*RAxML Randomized Axelerated Maximum Likelihood, *PhyML *Phylogenetic estimation using (Maximum) Likelihood, *MECAT *Mapping, Error Correction and de novo Assembly Tools, *SOAPdenovo *Short Oligonucleotide Analysis Package de novo, *ABySS *Assembly By Short Sequences, *MaSuRCA *Maryland Super-Read Celera Assembler, *LACHESIS *Ligating Adjacent CHromatin Enables Scaffolding In Situ, *OMGS *Optical Map-based Genome Scaffolding, *SNAP *Single Nucleotide Polymorphism Annotation Platform, *BLAST *Basic Local Alignment Search Tool, *BWA-mem *Burrows Wheeler Aligner Maximal Exact Match, *HISAT *Hierarchical Indexing for Spliced Alignment of Transcripts, *NGMLR *coNvex Gap-cost alignMents for Long Reads, *BLASR *Basic Local Alignment with Successive Refinement, *LASTZ *Large-Scale Genome Alignment Tool, *SyRI *Synteny and Rearrangement Identifier, *SVMU *Structural Variants from MUmmer, *SV *Structural Variants, *GATK *Genome Analysis ToolKit; *NGSEP *Next Generation Sequencing Experience Platform, *SURVIVOR *StructURal Variant majorIty Vote, *PSVCP *Pangenome construction and SV genotype Calling Pipeline, *PAV *Presence Absence Variation, *JASMINE *Jointly Accurate Sv Merging with Intersample Network Edges, *VCF *Variant Calling Format, *GFA *Graphical Fragment Assembly, *SNP *Single Nucleotide Polymorphism, *INDELs *INSertions and DELetions, *VG *Variation Graph, *PGGB *PanGenome Graph Builder, *BANDAGE *Bioinformatics Application for Navigating De novo Assembly Graphs Easily, *PANACHE *PANgenome Analyzer with CHromosomal Exploration, *ODGI *Optimized Dynamic Genome/Graph Implementation

#### Gene-based pangenomes

A gene-based approach to pangenome construction compares the number of annotated genes, families, and/or gene loci in chromosome level or allele aware annotated genome assemblies based on homology and synteny searches and returns the core and dispensable gene families among the genomes included in the analysis (Fig. 1a). However, this method compares only the genic portion of the genome and ignores the non-genic components. Various software used to develop a pan-gene database are mentioned in Fig. 3. Software tools such as OrthoMCL [188], Orthofinder [187] or GET_HOMOLOGUES-EST [97] can be used for these analyses. Protein sequence files are used as input from each of the genomes as demonstrated in pearl millet [39] and the maize [6]. To minimize bias, all genomes being compared should be annotated using same approach i.e., same gene prediction software and function databases. The number of genes included in a pangenome by this method depends on the percent sequence coverage and identity threshold values used in the homology searches to identify orthologs. Therefore, such values should be chosen carefully to separate the core and dispensable genes with high confidence.

Lin et al. [98] conducted comparative genomic analysis using two *Brassica rapa* double haploid and inbred cultivars using the gene-based pangenome approach. The whole genome sequence of two cultivars were assembled *de novo* using short read next generation sequencing technology. Then they re-annotated the already assembled and annotated *Brassica rapa* ‘Chiifu’ genome using same annotation software and resources as the other two genomes under study. They identified 63% complete gene models and 87% exons as core among the three cultivars. Before reannotation of the reference ‘Chiifu’ genome, 41.8 and 46.2% of the gene models in other two genomes appeared to be different as compared to this reference genome based on genomic variation detection using the cortex_var software suite [217]. After re-annotation, only 6.1 and 5.6% genes were different in the two genomes with respect to the reference. This report clearly describes the importance of using the same annotation methods for making fair comparisons between genomes.

Another method for genic comparisons is to use RNA-seq or transcriptome data which focuses on the functional implications of genetic variations. In the pangenome developed for maize [61], transcript level variation was evaluated with RNA-seq data from seedlings in 503 diverse maize inbred lines. They found an 82.7% dispensable genomic fraction represented by high confidence transcript assemblies. Importantly, by mapping short read transcript data to the reference genome and producing a *de novo* joint assembly of unmapped reads from all seedlings, novel transcripts were identified and the single genome reference bias in mapping studies was removed.

A third method for gene-based pangenome development is to use iterative pairwise collinearity analysis from annotated gene sequences. The MCScan software can be used for such analysis in which genes are added from each genome in a stepwise manner. If no collinear gene is present in the preceding step, then that gene is assigned a new locus. This method was utilized by Qin et al. [218] to develop the rice pangenome. Other approaches to generate gene-based pangenomes include annotating map-to-pan and iterative assembly-based pangenomes (explained in the next section) and comparing genes using above mentioned approaches.

Glick and Mayrose [219] compared these three approaches- *de novo*, map-to-pan, and iterative assembly- for their ability to identify core and dispensable genomes. They reported that the de novo approach identifies more novel genes compared to the other two methods. However, the *de novo* and map-to-pan approaches are expensive because they require high quality whole genome sequences and annotations for every individual. For pan genomes involving a large number of accessions, the iterative assembly approach is often more practical.

#### Whole genome sequence-based pangenomes

Since most of the genome in many crops is comprised of non-genic components such as repetitive elements (Table 1) and their role in structural and regulatory processes has been well established [220–223], comparisons of non-genic sequences are also important. The different methods are compared in Figs. 1b and c, and 2. Ideally, all the genomes being compared should be of high quality and sequenced using same technology to remove any potential bias towards one genome. The accurate sequencing and assembling of large and repetitive crop plant genomes is challenging and expensive, yet it is still the preferred method for pangenome development. For more comprehensive and contextual biological analysis, it is a common practice to develop both annotated gene-based as well as whole genome sequence-based pangenomes. The crops in which both gene- and sequence-based comparisons were used include *Glycine soja* [103], *Oryza sativa* [104], *Brachypodium distachyon* [67], *Medicago truncatula* [60], and most of the pangenome analyses published after 2017. Whole genome sequence-based approaches to pangenome creation can be further subdivided into linear- and graph-based methods.

##### Linear pangenomes

Linear sequence-based approaches include map-to-pan and iterative assembly approaches. The flowchart explaining the available pipelines is shown in Fig. 3. These pipelines are discussed in the next section.

**Map-to-pan approach**

In the map-to-pan method, each genome is sequenced and assembled using *de novo* assembly software to the pseudomolecule/chromosome scale, and then genome-wide synteny is evaluated by making pairwise comparisons to discover core and dispensable genome fractions with reference to a single high-quality genome. To create a linear pangenome, pairwise whole genome alignment and a long- read aligner tools such as MUMmer [159], minimap2 [156], or NextGenMap-Long Reads (NGMLR) [157] are used, which identify the SNPs and small (< 50 bp) and large (> 50 bp) SVs in the genomes. Other short read and/or multiple sample alignment tools are mentioned in Table 3. For making unbiased comparisons, the reference genome is re-sequenced using the same *de novo* approach as other genome assemblies as demonstrated in *Glycine soja* [103], *Oryza sativa* [104, 224], *Medicago truncatula* [60], *Solanum lycopersicum* [69], *Sesame indicum* [109], *Pisum sativum* [68], and *Gossypium* sp [75].

A linear pangenome approach, called the Pangenome construction and SV genotype Calling Pipeline (PSVCP), was developed in rice by Wang et al. [191]. This method retains the positional information of genetic variants within a linear pangenome to facilitate downstream analyses such as association analyses. First, a linear pangenome is constructed with a map-to-pan approach using MUMmer [159] and all the variants are inserted back into the pangenome at positions determined by Assemblytics [167]. The pangenome is then used as a reference to align short reads for identifying PAVs, translocations and inversions by analyzing the mapping coverage at the breakpoints.

**Iterative assembly approach**

The iterative assembly approach can be used when the whole genome assemblies are highly fragmented, which is usually the case in highly heterozygous, cross pollinated crop species. In this approach the whole genome short and long read sequencing data are mapped directly to a single high-quality reference genome assembled at a chromosome level. Then, un-aligned reads are re-assembled using a *de novo* approach with software tools such as Ray [133] and MEGAHIT [134]. The newly assembled contigs are added back to the reference genome. These newly assembled contigs represent the missing fraction in the original single reference. Depending on the number of genotypes used, if only a few genotypes are involved, re-assembly can be performed individually for each genome. However, with a larger number of genotypes, unmapped reads from all genomes can be pooled together for re-assembly. An important consideration in the latter approach is to remove the redundant sequences from the pooled contigs using nucleotide sequence- based clustering tools such as CD-HIT [190] or BLASTx against National Center for Biotechnology Information (NCBI) Non-Redundant (NR) proteins or a similar database. Again, care should be taken to use high similarity thresholds, identity values, bit-score, and alignment length cut-offs to keep high confidence non-redundant sequences to add in the pangenome. To find the location of the newly added contigs in the original reference, researchers [88, 89] have utilized a linkage disequilibrium approach by identifying SNPs on these contigs in high linkage disequilibrium with SNPs on the original reference genome. This iterative assembly approach has been widely used in many crops such as wheat [66], rice [84, 105], rapeseed [90, 225], pepper [87], sunflower [88, 226], pigeon pea [99], sorghum [85, 110], pea [68], chickpea [89], and mungbean [56]. For cases where sequencing depth is low (< 10x), the Eukaryotic Pangenome analysis toolkit (EUPAN) [227] can be utilized as a map-to-pan automated pipeline. Another metagenome-like pangenome method has been also used in rice [62]. In this approach, short or long sequencing read data with sequencing depth ranging from 1 to 3x from > 700 individuals were pooled and assembled using a *de novo* approach, then compared with a reference genome assembly.

These linear sequence-based approaches will generate a pangenome containing the missing sequences to represent the diversity of many individuals, but they will not preserve the coordinate system of the original reference genome. It will be difficult to find the location of previously mapped genes in such a pangenome. Therefore, such pangenomes need to be re-annotated using information from individual assembly transcript data with various gene prediction approaches (see next section for more details). The graph-based pangenomes offer a solution to this difficulty.

##### Graph-based pangenomes

Broadly two types of approaches have been used to build graph-based pangenomes (Figs. 2 and 4). The first approach uses a reference genome as a backbone and compares it with other genomes to identify SNPs, INDELs, and SVs. Multiple tools have been developed for creating graph-based pangenomes and the most commonly used pipelines for reference backbone pangenome graphs in eukaryotic genomes are Variation Graph (VG) Toolkit [192] and minigraph [194]. The second approach to build a graph pangenome does not rely on a reference genome, and as such is a truly reference free approach. The two reference free pipelines are Minigraph-Cactus [195] and PanGenome Graph Builder (PGGB) pipeline [196]. All the above- mentioned approaches are called Variation graph approaches. Another approach which is reference free is a k-mer based color compacted de Bruijn graph approach. These methods are discussed in the next sections.

**Reference backbone graph pangenomes**

The graph-based pangenome methods are very similar to the variant calling format (VCF) process. In this approach, positions of SNPs and SVs are marked on the original reference genome as nodes, keeping the base reference genome coordinate system intact. Simply, it is a VCF file represented in the form of a graph. The first step to construct a graph-based genome is to identify all forms of genetic variation such as SNPs and SVs among a group of genomes. A VCF file and a reference genome is used as input in the ‘vg construct’ utility of VG toolkit to output a graph pangenome in VG format. Therefore, other methods or software tools must be used to identify the genetic variation to create the VCF file. This can be done by using whole genome alignment tools such as MUMmer [159], AnchorWave [160], or minimap2 [156]. The all-vs-all pairwise whole genome alignments can further be used to identify SVs using tools such as Synteny and Rearrangement Identifier (SyRI) [165] for variants > 50 bp as well as Structural Variants from MUMmer (SVMU) [166] and Assemblytics [167] for variants of all sizes. Short read data can also be used to detect SVs using smartie-SV [168], PBHoney [170], and NanoSV [171]. The methods and algorithms for structural variant detection have been comprehensively reviewed by Ho et al. [228] and Kosugi et al. [229]. The non-redundant SVs can be combined using the merge function in the bcftools package. Other software for merging the SVs across different whole genome sequence comparisons include SURVIVOR [183] and JASMINE [186]. PanPop [184] offers an advantage by efficiently merging SVs based on both short-read data and whole-genome sequences across big populations, where SVs information get complex, by leveraging both sequence and positional similarity. The crops in which a graph-based pangenome has been developed using VG Toolkit include soybean [102], rice [111, 218], radish [108], cucumber [101], pepper [100], tomato [230] and foxtail millet [231].

Another graph-based method uses the minigraph [194] pipeline in which whole genome sequence alignments of multiple genomes, one of them marked as the reference genome, can be used as input files to generate a pangenome graph. This workflow is an iterative map-to-pan version of graphical pangenomes, and is, basically, an iterative sequence-to-graph method. It uses minimap2, a pairwise whole genome sequence aligner, and outputs a pangenome in reference graphical fragment assembly (rGFA) format in which position of any segment can be traced back to its original genome. The whole genome assemblies being used to generate a graph should be similar to each other, ideally belonging to the same species, for accurate results. The minigraph pipeline does not work well with more highly diverged genomes and it only includes variations > 50 bp in the final graph. Recently, the minigraph pipeline was used in rice [106] to develop a graph-based pangenome.

**Reference-free graph pangenomes**

The Minigraph-Cactus [195] is another same species pangenome construction method that takes advantage of the fast graph construction algorithm of minigraph. It uses a modified version of Progressive Cactus [161] as a base aligner to develop guide tree free alignments in hierarchical alignment (HAL) format and outputs the pangenome in other multiple standard formats such as VG and rGFA. Haplotype-resolved assemblies can be used to construct a pangenome using this pipeline. This method includes variation of all sizes in the final graph.

Another reference free pipeline available for graph pangenome building is PGGB [196]. It uses an all-to-all alignment of the assemblies. It outputs the file in GFA 1.0 format in which the position of the DNA segment in the graph cannot be traced back to its linear genome used to build the graph. It takes a very long time to run for large genomes, so it is advised to split the genomes into chromosomes and run the analyses for each chromosome separately. Like Minigraph-Cactus, it also includes all sizes of variations in the graph and produces very complicated graph structures at diverse loci. Briefly, the PGGB pipeline uses multiple other software and is a three- step process: (1) it produces all-to-all alignments using wfmash aligner [232]; (2) then it uses these alignments to build the graph in GFA v1.0 format with seqwish [233] and; (3) lastly, it uses a normalization algorithm in smoothxg to simplify complex repetitive regions and removes the redundant regions using GFAffix.

Cuttlefish [197] and Bifrost [198] are other programs that can be used to build a reference free pangenome. These are based on a k-mer method used to build de Bruijn graphs. These programs are much faster than the variation graph approaches mentioned above. They also store all types of variations but extraction of information from these graphs is challenging due to the size of the output files which includes overlapping DNA sequences (the k-1 overlaps between nodes) of size k. Also, selecting an appropriate k-mer size for highly diverse genomes is challenging. Selecting the size of k-mers significantly impacts the complexity of the graph. There isn’t an ideal selection because as the k-mer size increases gradually, the de Bruijn graph becomes less intertwined but more fragmented. For downstream analyses in k-mer based graph pangenomes, Cleary et al. [234] developed a software called FindFRs. They define frequent regions (FRs), as regions in the graph that are traversed by many sequence paths. These FRs represent the inexact syntenic regions between different haplotypes in the graph. As a test case, FRs were identified in a yeast pangenome graph developed using Cuttlefish [197]. These paths and the number of times each region was traversed were used as features in support vector machine models to categorize yeast genomes into different classes. Examinations of the FRs in yeast uncovered introgressions which contribute to alcohol tolerance, confirming their biological significance. Furthermore, FRs can be used for categorizing yeast strains based on industrial applications and visualizing the pangenomic landscape. It remains to be seen how well this software performs in larger, more complex plant genomes.

Andreace et al. [235] compared different methods of creating graph pangenomes for stability, editability and scalability using human genome assemblies. They reported that Bifrost produces the most stable graph when run multiple times using same input. Minigraph produces a slightly different graph if the order of the input sequences is varied. Minigraph-Cactus and PGGB produce slightly different outputs when run multiple times using identical input. Out of all these tools, minigraph requires the least run time, followed by Minigraph-Cactus, PGGB, and then de Bruijn graph- based approaches. Bifrost and Cuttlefish lack elaborate tools for downstream analyses, but other approaches can be easily used for mapping, variant calling etc. as shown by Liao et al. [236].

An important issue with the graph-based pangenomes is the compatibility of various graph-based pangenome formats, such as GFAv1.0, GFAv1.1, rGFA, VG, and HAL, with different tools (Fig. 4 – Downstream Analyses). Each pipeline has its own method for describing paths and nodes in the graphs, which complicates interoperability. For instance, the Minigraph rGFA format cannot be used with many VG tools, like VG augment, even after conversion of ‘GFA’ to ‘VG’ format using ‘vg convert.’ While several sequence-to-graph alignment tools have been developed, they are highly specific to certain formats, and none of them are compatible with all pangenome formats.

### Challenges of pangenome annotations

Once a pangenome is developed, it should be annotated with the positions of all genes and repetitive elements, and function should be assigned to each element. The first approach for structural annotation of a linear pangenome is to annotate each genome individually and combine the results of each genome to develop a gene-based pangenome using pairwise collinearity analysis. Various software tools such as MAKER_2_ [145] and BRAKER_2_ [146] are available to conduct transcript evidence-based gene predictions in a single genome using RNA-seq and Iso-seq data. The resulting gene annotations are used to train parameters for ab initio gene prediction models using software like AUGUSTUS [237], GeneID [238], GlimmerHMM [149], and SNAP [150] which can be combined at a later point. These trained models are used for novel gene predictions. Functional annotation is done using publicly available protein databases either for a specific crop or for less resourced organisms, universal gene banks for plants such as InterProScan 5.0 [74], UniProtKB/Swiss-Prot and UniProtKB/TrEMBL [239], the Arabidopsis Information Resource (TAIR) [240], Uniprot100 [241], NCBI-NR protein database, iTAK [242], PlantRegMap [243], and pfamA [244].

The repetitive fraction of the genome can be characterized to identify various types of transposable elements (TEs). Repeat sequences in the newly assembled genome can be identified and annotated using Extensive *de novo* Transposable Elements (TEs) Annotator (EDTA) [245], which is composed of eight published programs including LTR_Finder [246], LTRharvest [247], and LTR_retriever [248] to identify long terminal repeat (LTR) retrotransposons. Generic Repeat Finder [249] and TIR-Learner [250] are included to identify TIR transposons; HelitronScanner [251] identifies Helitron transposons. RepeatModeler [252] is used to identify TEs such as SINEs and LINEs missed by the other structure-based programs. The RepeatMasker [253] can be used to annotate fragmented TEs based on homology to structurally annotated TEs. Also, Tandem Repeats Finder [254] can be used for identifying tandem repeats. These software tools and prediction algorithms can be used for identifying the different repetitive elements and TEs in the genomes individually. The results from each genome can then be combined to identify the pan-TEs using the panEDTA [245] pipeline.

Another approach for structural annotation is to develop the linear pangenome first and then annotate the dispensable fraction using the same gene prediction models which were used to annotate the individual genomes, as demonstrated in rice [84, 105], cabbage [225], tomato [69], and pigeon pea [99]. This is helpful if the pangenome is closed i.e., no further significant increase in the size of core and dispensable fraction is expected. As mentioned earlier, this process will disrupt the gene coordinate system of the genomes and new positions will be assigned to all the genes. As a consequence, it is difficult to determine the chromosomal position of genes in the dispensable fraction of the pangenome. However, new chromosomal positions can be assigned to some of the added contigs using partially mapped read pairs as done by Golicz et al. [225] and Hübner et al. [88]. For the sunflower pangenome [88] and maize pan-transcriptome [61], researchers used an LD mapping approach to find the genomic position of *de novo* assembled contigs or genes which are absent in the reference genome. In this approach, LD is calculated between new contigs and SNPs of known genomic location on the reference using an association analysis model in which a gene presence-absence matrix is used as the phenotype and the SNPs are used as genotypes. A few studies, such as those by Hurgobin et al. [90] and Ou et al. [245], have annotated the pangenome with MAKER_2_ using transcript evidence provided by RNA-seq and Iso-seq data from all individual samples.

Methods available to annotate a graph-based genome are limited and are still in their infancy. In a graph-based pangenome, annotations are added to the graph pangenome using the coding sequences and annotation files developed for individual genomes. In tomato [228], the minigraph in-built sequence-to-graph mapping function was used to map the coding sequences against the graph for annotation. Some examples for graph annotation tools include graph-gene-caller (ggCaller) [208], and PanTools [209, 210]. The VG Toolkit offers a ‘vg rna’ utility in its pipeline to generate an annotated graph-based pangenome called a ‘spliced pangenome graph’ constructed by using reference genome annotations. Also, a haplotype aware annotated graph called a ‘graph-based pan-transcriptome’ can be constructed by adding annotation information from all the genomes using ‘vg rna’ tool. Additionally, haplotype specific transcript expression quantification can also be performed. For this, ‘vg mpmap’ is used to align RNA-seq reads to these graph-based pan-transcriptome. Lastly, RPVG [211], an independent tool, can use the alignments from ‘vg mpmap’ to quantify haplotype-specific transcript expression. This approach has been applied in human genomes and has not been tested in any crop species.

### Implications of the pangenome

The core genes are mainly involved in fundamental biological, molecular, and cellular functions. The dispensable fraction of the genome in many crops includes genes involved in important agronomic traits such as biotic and abiotic stresses response [61, 62, 66, 112, 225]. Also, dispensable genes are more likely to lack a known function and are typically expressed at lower level or not at all [61, 71]. TEs play an important role in genome evolution and dispensable genes present in one to two individuals are often found to be enriched with TEs [70–72, 112, 225]. Pangenome analyses in *Brachypodium distachyon* [67] and *Arabidopsis thaliana* [97] has revealed that the evolutionary rate of dispensable genes is higher than that of core genes. These dispensable genes are expected to be under relaxed purifying selection with higher non-synonymous substitution rates. Additionally, these genes show high CNVs in kinases and RNA recognition domains.

In dispensable genes, the type (SNPs vs. CNVs) and range (large vs. low effect) of SNPs and SVs also differ among various gene families. Zhou et al. [60] compared multiple gene families including nucleotide-binding sites – leucine rich repeats (NBS-LRRs, ) heat shock proteins (HSPs), LRRs, receptor-like kinases (RLKs), F-box proteins, and NCRs in the *Medicago truncatula* pangenome for differences in variant architecture. In the dispensable part of the *M. truncatula* pangenome, the NBS-LRRs and HSPs were found to be the most variable gene families containing a high frequency of large effect SNPs. On the other hand, RLKs, F-box-proteins and NCRs were less variable and less frequently present in the dispensable genome.

Hirsch et al. [61] developed a transcript assembly representing a pan-transcriptome by incorporating the unmapped (to the reference B73 genome) RNA-seq reads from 503 diverse maize inbred lines. Then, RNA reads from B73 were mapped to this transcript assembly. They identified 4,341 novel expressed transcripts in B73 mapping to this pan-transcriptome, which were previously thought to be absent in this cultivar. In rice [218], 17.5% of SVs were not linked with SNPs indicating a significant reservoir of genetic diversity that cannot be captured using SNP markers [218]. In multiple other species such as pigeon pea [99], pearl millet [39, 68, 99] and garden pea [68], SVs were found to be associated with agronomic traits resulting in QTLs which were not observed in SNP based QTL and GWAS studies. This suggests that previously published short read data can be reanalyzed using pangenomic tools to identify important new variants and marker-trait associations.

With the advancement in long-read sequencing technologies, it is now possible to sequence and assemble all homologs, even in polyploid species [34, 35]. These haplotype aware assemblies can be used to study the differential loss of genes between haplotypes, which often is the result of whole genome duplication events [255]. Before haplotype aware assemblies, we relied on consensus sequences with a single allele per locus, developed from inbred lines or monoploids. The drawbacks of monoploid genome assemblies are particularly acute in polyploids because polyploids have more alleles per gene than diploids [256]. For example, the number of genes captured in allele-aware assemblies in alfalfa [34, 35] is much higher compared to the consensus monoploid ZhongmuNo.1 [33] assembly. Notably in alfalfa, not only is the number of total annotated genes using Iso-seq isoforms and RNA-seq data higher in the allele-aware assemblies than monoploid assembly, also, the percentage of genes functionally annotated using databases such as NR, GO, KEGG, Swiss-Prot, and TrEMBL in XinJiang DaYe is 96.5% (158,894 of 164,632) compared to 77% (37,885 of 49,165) in ZhongmuNo.1.

Allele-specific sequence information is especially important when designing gene editing using systems like CRISPR/Cas [35]. However, there are currently no linear read alignment software programs which can effectively utilize all haplotypes of a genome assembly as a reference. Researchers must either choose one of the haplotypes or the longest haplotypes (grabbing the longest chromosome for each homolog copy) from the allele-aware assemblies to use as the reference in mapping and association studies. The closest approximation is the Practical Haplotype Graph [257], which offers variant imputations based on stored haplotype information from a pangenome for studies with large amounts of missing data such as genotyping-by-sequencing. A graph-based pangenome effectively captures multiple haplotype information from many cultivars. The VG Toolkit can be used for short read mapping (vg giraffe, map) and variant calling (vg call) directly to a graph-based pangenome. Minigraph sequence-to-graph mapping is another option to map a gene sequence against the graph pangenomes. Other tools such as GraphAligner [201] and GraphMap [200] can be used to align long reads to a graph pangenomes. Different variant callers for graph based pangenomes include ‘vg call’ utility in VG Toolkit (only works with VG format), GraphTyper [204, 205], Paragraph [203], BayesTyper [207], and PanGenie [206].

Transitioning from employing a single genome to a pangenome as a reference is expected to enhance variant calling accuracy and facilitate the identification of genes linked to important traits. In humans, mapping short-read data against the recently developed draft pangenome resulted in 34% fewer errors detected in variant calling than mapping the same reads against the linear GRCh38 human reference genome [236]. Additionally, using the pan genome led to a 104% increase in the identification of SVs per haplotype compared to using the single reference. In tomato, the graph pangenome has been shown to be more effective than the linear genome in detecting all types of genetic variants. Linear pangenome yielded only 20% of the SVs called by the graph pangenome [230]. Additionally, they reported that average heritability estimates obtained using the graph pangenome were higher than those derived from the linear reference genome. Mapping studies using a pangenome as a reference have been carried out in *pearl millet* [39] and tomato [230] with VG Toolkit, and in pigeon pea [99] using a linear pangenome developed with an iterative mapping approach.

### Alfalfa pangenome – a case study

Developing a pangenome for a polyploid and highly heterozygous crop species with a large genome size such as alfalfa (2n = 4x = 32; genome of ~ 800 Mbp) is especially challenging and costly as it requires many genomes to be sequenced and assembled accurately. The development of high-fidelity Pac-Bio sequencing, Hi-C technology, and Bionano optical maps have greatly facilitated development of haplotype resolved assemblies. Polyploids tend to be more heterozygous than diploids due to the number of chromosomes [258] and weakened selection [91] thus amplifying the challenges associated with highly heterozygous assemblies [259] particularly for out-crossing species. The autotetraploid alfalfa has higher average heterozygosity (3.7%) compared to diploid alfalfa (1.9%) [35, 260], which can result in allelic haplotypes being scaffolded into chimeric contigs [255]. These chimeric contigs in the assembled genome can be identified using Hi-C technology and genetic linkage maps. Therefore, much more manual curation is needed for assembling a genome in outcrossing polyploids compared to diploids. Additionally, when consensus monoploid genome assemblies are produced in autotetraploid cross-pollinated species, heterozygosity can result in highly fragmented assembled genomes [45]. In addition to the challenges of *de novo* assembling an autotetraploid genomes, the cost required to produce a high-quality assembly is also increased due to the high coverage and sequencing depth required to assemble autopolyploid genomes. Accurately distinguishing between heterozygous classes in a polyploid requires 60X coverage [261]. Furthermore, this increases the number of individuals needed to capture the total range of haplotypes in a pangenome. The polyploid species where a pangenome has been developed include wheat [66], cotton [75], rapeseed [90] and potato [70, 71].

Many different tools and pipelines have been developed or are currently being developed for constructing, visualizing, and using pangenomes in crop species. These tools and a step-wise process are summarized in Table 3. Recently, one consensus chromosome scale [33] and two haplotype aware chromosome scale [34, 35] genome assemblies of alfalfa cultivars from China have been published. Another accession of cultivated alfalfa at the diploid level (CADL) [262] from United States germplasm has been sequenced with 5,753 contigs. These assemblies were compared using pairwise comparison with linear aligners and graph-based pangenome methods available for this species. Alfalfa has been grown in China for millennia, largely independent of outside influence, resulting in distinct genetic traits [263, 264]. The three tetraploid cultivars with chromosome scale assemblies have the same geographical origin, and therefore we expect they encompass only a fraction of the overall genetic diversity in alfalfa. This analysis is presented to showcase the high amount of variation that is present in only three genotypes of alfalfa grown in China.

#### Pairwise comparison of three alfalfa assemblies

To identify structural variations using the pairwise alignment comparison, all four genome assemblies were used. MUMmer (v. 4.0.0beta2) was used, with following parameters: -c 500 -t 50, to make pairwise comparisons to other three genomes with ZhongmuNo.1 as the reference. The filtered delta file using ‘delta-filter -m -i 90 -l 100’ options for each pair of comparisons generated was used as the input in the browser version of Assemblytics [167] and parsed to detect variants > 50 bp and < 10,000 bp. The lowest number of SVs were observed for the CADL genome. Notably, the CADL assembly is not contiguous, but highly fragmented compared to the other three genomes, and therefore, we might not have captured all the SVs. Additionally, the CADL assembly belongs to a diploid plant which may have resulted in identification of fewer SVs compared to other two haplotype-aware autotetraploid genomes as there are fewer alleles per locus. In three pairwise comparisons – (a) ZhongmuNo.1 vs. XinJiang DaYe, (b) ZhongmuNo.1 vs. ZhongmuNo.4, and (c) ZhongmuNo.1 vs. CADL, the total number SVs representing insertions and deletions were 35,823, 37,532, and 20,064, respectively. Out of total SVs detected in these three comparisons 63.3, 61.8, and 63.6% belongs to repeat elements expansion and contractions, respectively for three pairwise comparisons. In a similar study conducted in *Medicago truncatula* [265], a diploid relative of alfalfa, using two genome sequences – ‘Jemalong A17’ and ‘R108’, it was observed that 82.53% of SVs were located within repeat regions. Alfalfa has five times more TEs compared to *M. truncatula* [33]. This observed difference in SVs may be attributed to the polyploid and cross-pollinating nature of alfalfa versus diploid and the self-pollinating nature of *M. truncatula.* Similar results were found in a study on potato [266], where a pangenome constructed from a limited set of monoploid and doubled monoploid clones revealed a greater extent of structural variation compared to diploid, sexually reproducing potato. This suggests that the size of a species’ pangenome is influenced by factors such as reproductive mode, ploidy level, and the genetic diversity sampled. The only autotetraploid species where a pangenome has been developed is potato [70, 71, 266]. In the study by Bozan et al. [70], a linear pangenome was constructed using 296 diverse potato and wild relative accessions with different ploidy levels. An additional 7.3 GB of sequences were integrated into the reference genome, of which approximately 5.9 GB (79.5%) consisted of repetitive elements. The results in alfalfa for SVs in repetitive regions could be an underestimation because the three genome assemblies used in this analysis were not assembled and scaffolded using the same sequencing technologies. However, all three genomes are high quality genomes based on the Benchmarking Universal Single-Copy Ortholog (BUSCO) [267] assessment results which were 93.3, 98.4, 97.2, and 96.7% for the ZhongmuNo.1, ZhongmuNo.4, XinJiang DaYe, and CADL genome assemblies, respectively.

The filtered coords output files from MUMmer, processed with the ‘show-coords -THrd’ options, along with filtered delta file, were used as input for the Synteny and Rearrangement Identifier (SyRI v.1.6.3) [165] to identify all types and sizes of genomic variants between ZhongmuNo.1 and homologs from two other chromosome-level genome assemblies, XinJiang DaYe and ZhongmuNo.4. The results are summarized in Table 4. Interestingly, most of the regions affected by SVs are attributed to inversions, translocations, and duplications, impacting regions ranging from 56.32 to 193.27 MB, 126.90 to 163.54 MB, and 181.37 to 211.39 MB, respectively, in ZhongmuNo.1. The variants from all homologs of two assemblies were merged and normalized using bcftools v.1.16, resulting in the identification of 71,635,059 genomic variants, including 59,498,682 SNPs, 6,965,934 insertions, and 5,170,443 deletions.

**Table 4:** Genomic variations identified by Synteny and rearrangement identifier (SyRI) in the ZhongmuNo.1 alfalfa assembly

Variation Type	XJDY-homolog1^a^		XJDY-homolog2		XJDY-homolog3		XJDY-homolog4		Zm4-homolog1		Zm4-homolog2		Zm4-homolog3		Zm4-homolog4	
	MB^b^	Count	MB	Count	MB	Count	MB	Count	MB	Count	MB	Count	MB	Count	MB	Count
Syntenic Regions	280.39	50,832	275.43	51,949	282.65	52,462	276.54	49,978	178.68	39,606	160.32	36,991	147.95	31,403	144.33	32,086
Inversions	77.47	746	79.76	812	56.32	801	73.79	772	193.17	1,150	176.96	1,145	187.42	1,141	189.74	1,092
Translocations	126.90	44,674	133.17	46,877	137.02	47,841	128.89	45,145	163.54	42,281	158.32	43,966	156.84	37,666	148.95	39,342
Duplications	183.27	73,767	183.63	74,509	187.90	75,062	181.37	73,834	201.52	84,661	206.22	91,823	211.39	90,283	203.84	91,524
Insertions	15.46	3,553,038	16.18	3,666,861	14.80	3,587,399	15.23	3,534,034	23.74	3,950,392	23.01	3,858,363	28.59	3,725,835	21.06	3,609,468
Deletions	16.00	1,333,727	14.69	1,370,368	13.70	1,365,463	13.91	1,328,741	23.78	1,390,132	22.71	1,363,097	24.72	1,292,639	22.56	1,262,327
SNPs^c^	13.33	13,330,641	13.83	13,826,444	13.60	13,598,133	13.39	13,387,943	13.47	13,469,161	13.39	13,394,735	12.62	12,624,244	12.41	12,405,676
Not Aligned	181.89	96,952	181.82	99,109	190.91	100,860	190.97	97,172	140.24	93,663	165.66	101,031	162.98	93,943	173.15	97,913

^a^XJDY refers to ‘XinJiang DaYe’ and Zm4 to ‘ZhongmuNo.4’, two allele aware alfalfa genome assemblies with four homologs each. Each homolog was used in pairwise comparison against ZhongmuNo.1 assembly

^b^MB: Million Bases

^c^SNPs: Single Nucleotide Polymorphism

**Fig. 5:**
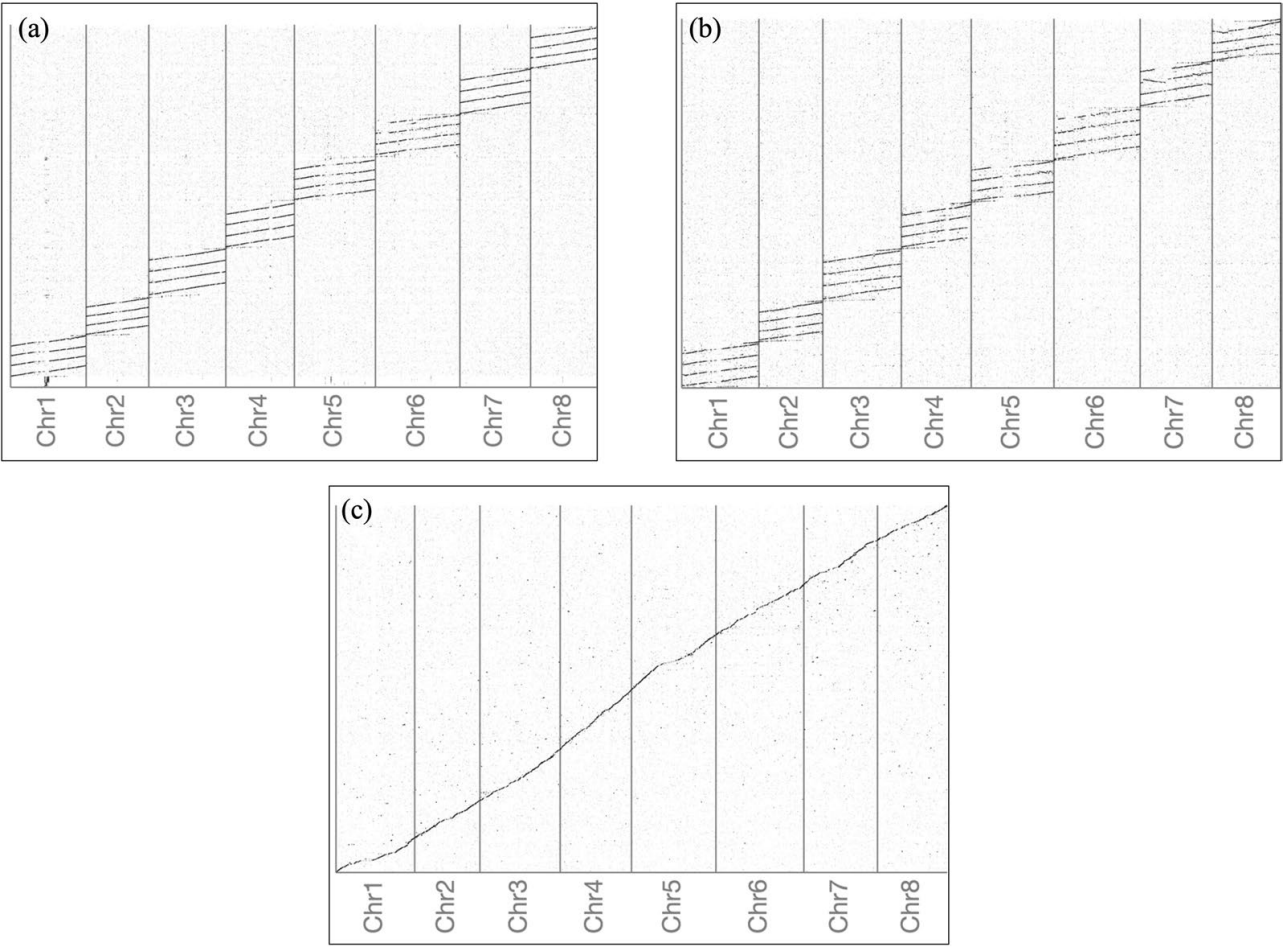
Dotplots comparing Medicago sativa assemblies. The ZhongmuNo.1 consensus genome assembly (y-axis) is comapred to the **a** XinJiang DaYe, **b** ZhongmuNo.4, and **c** CADL genome assemblies. Nucleotide level alignments were generated using MUMmer (v. 4.0.0beta2). Dotplots showing unique alignments were generated using web version of Assemblytics

The common structural variants (SVs) of size at least 50 bp, identified by both SyRI and Assemblytics, were combined using SURVIVOR v1.0.7 [268] with the following parameters: ‘merge 1000 2 1 1 0 50’. This provided 41,590 SVs in the ZhongmuNo.1 genome assembly with respect to the two other allele-aware assemblies. The 44,697,006 biallelic SNPs and 41,590 SVs were annotated using SnpEff v.5.2c [269] software (Table 5). Based on the annotation results, an interesting observation is that 50.1% of SNPs lie outside the genic region, compared to just 33.0% of SVs. Additionally, 13.61% of SVs were found to have a high impact on genes, leading to effects such as protein truncation, loss of function, or codon change-mediated decay. In contrast, only 0.10% of SNPs were classified as having a high impact on genes. There was one SNP every 17 bp and one SV every 19,000 region. Unlike SNPs and INDELs, SVs are believed to have a more significant impact on gene expression and protein function [270]. The high impacted gene variants were enriched with Gene Ontology (GO) terms using the topGO v2.54.0 R package. The top 10 GO terms for biological processes, cellular components, and molecular functions are presented in Fig. 5. The GO term results for SNPs and SVs were similar, except for the molecular functions, indicating that both types of genomic variants affect similar regions in the alfalfa genome. The biological processes and cellular component terms for both SNPs and SVs include ‘response to hormone’, ‘response to chemical’, ‘response to endogenous stimulus’, ‘protein containing complex’, ‘extracellular region’ among others. The top GO terms in molecular functions for SNPs include molecular function and enzyme inhibitor and regulatory activities, whereas, for SVs it includes ‘ADP binding’ and ‘RNA-dependent RNA polymerase activity’ among others.

**Table 5:** Annotation of genomic variations identified in ZhongmuNo.1 alfalfa genome reference assembly

Type^a^	SNPs (SyRI)		SVs (SyRI and Assemblytics)	
	Count	Percent	Count	Percent
Downstream	15,444,954	20.281	26,451	23.134
Exon	2,928,069	3.845	10,566	9.241
Gene	0	0.000	803	0.702
Intergenic	38,195,887	50.155	37,705	32.977
Intron	4,044,287	5.311	6,047	5.289
Splice_Site_Acceptor	8,032	0.011	224	0.196
Splice_Site_Donor	7,037	0.009	271	0.237
Splice_Site_Region	97,626	0.128	442	0.387
Transcript	112	0.000	4,638	4.056
Upstream	15,152,074	19.896	26,792	23.432
UTR_3_Prime	170,191	0.223	267	0.234
UTR_5_Prime	106,932	0.140	132	0.115

^a^Annotation type of genomic variations categorized using SnpEff v.5.2c based on their positions in the annotated ‘ZhongmuNo.1’ reference genome. ‘Downstream’ represents variant located within 5 kb downstream from a gene; ‘Splice_site’ indicates a splice variant that changes the 2 bp region at the 3’ or 5’ end of an intron; ‘Transcript’ indicates a feature ablation whereby the deleted region includes a transcript feature; ‘Upstream’ indicates variant located within 5 kb upstream of a gene. The rest of the terms are self-explanatory

**Fig. 6:**
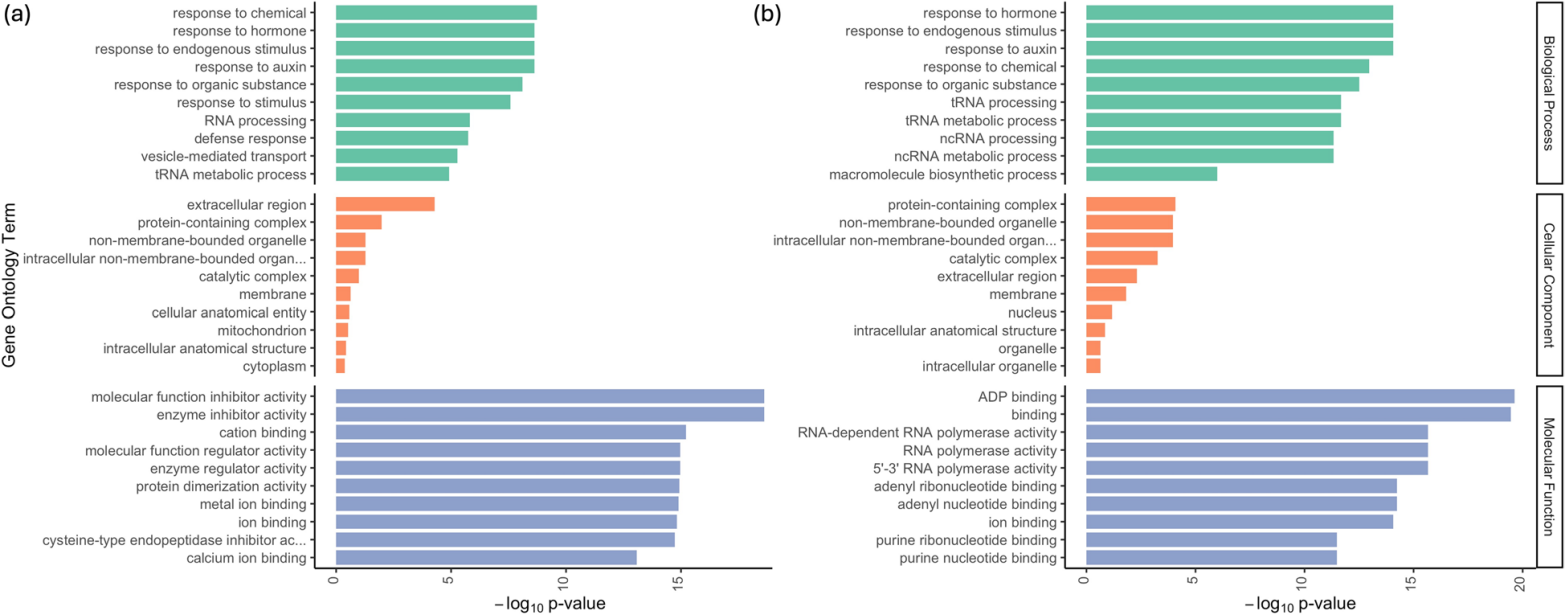
GO enrichment of the highly-impacted-by-variant genes. **a** SNPs identified in ZhongmuNo.1 reference genome using SyRI v.1.6.3 separately for each of the homologs for two reference genomes, ‘XinJiang DaYe’ and ‘ZhongmuNo.4’ and later combined using BCFtools v.1.16, **b** Structural Variants (< 50 bp) identified using SyRI v.1.6.3 and web version of Assemblytics separately for each homolog and later combined using SURVIVOR v.0.1.0

The nucleotide level unique alignments and dotplots were produced from all three pairwise comparisons at the whole genome level using the web version of Assemblytics (Fig. 6). Differences in coverage are clearly visible especially in chromosome 1, 5, and 6 in XinJiang DaYe and ZhongmuNo.4 (Fig. 6a and b) when compared with the ZhongmuNo.1 assembly represented on the y-axis. The CADL assembly, which comes from a diploid alfalfa plant, in contrast, shows better coverage of the ZhongmuNo.1 assembly (Fig. 6c). Also, we can notice the multiple inversions between all three comparisons in the dotplots (Fig. 6a and b, and c). Medina et al. [271] conducted a survey on the genetic diversity in alfalfa germplasm pools chosen from northern latitudes. They observed that the variance among individuals within populations is greater than the variance between populations. For a more comprehensive view of the alfalfa pangenome, analyzing more broad-based germplasm to capture the whole range genetic diversity is crucial.

#### Comparison of graph-based pangenome methods

For a graph-based pangenome, the base reference genome used was ZhongmuNo.1, a monoploid assembly of 794 Mbp with eight assembled pseudo-chromosomes (unplaced contigs were removed). The four haplotypes of the XinJiang DaYe and ZhongmuNo.4 assemblies were split into monoploid versions and added sequentially along with the CADL assembly to create the graph-based genome. The minigraph with ‘-cxggs -t16’ parameters and Minigraph-cactus pipelines with ‘--chop --giraffe --vcf --gfa --gbz –permissiveContigFilter’ parameters were compared by generating a reference graphical fragment assembly (rGFA). The results are summarized in Table 6. The minigraph pangenome has a length of 1.4 GB. Additional sequences of 0.61 GB were added in the ZhongmuNo.1 reference genome representing SVs among the four reference genomes. We noticed that input order of the genome sequences in minigraph is important, and results vary slightly for the number of nodes and links in the final graph if order is changed. For the Minigraph-Cactus pipeline, the pangenome length was 1.6 GB with 0.8 GB additional sequences added to ZhongmuNo.1 assembly. Minigraph-Cactus discovers all sizes of SVs and SNPs when assemblies are compared whereas, minigraph only identifies SVs larger than 50 bp. A third graph pangenome was generated with the PGGB pipeline with default parameters using the nextflow-pangenome tool [272]. For this analysis, each chromosome was treated separately including genome sequence from three assemblies representing nine haplotypes (one haplotype from monoploid ZhongmuNo.1 assembly, four haplotypes each from allele aware XinJiang DaYe and ZhongmuNo.4 assemblies). The total length of the PGGB pangenome evaluated using ‘gfatools stat’ was 2.91 GB, which is almost double compared to the two previous graphs. The CADL genome assembly was not included in the PGGB analyses as it is a fragmented assembly, and still it was the most complex graph out of three compared. The PGGB pipeline had the significantly longest runtime, followed by Minigraph-Cactus and Minigraph (Table 6).

**Table 6:** Comparison of three graph-based pangenomes development pipelines in alfalfa

Feature^a^	Minigraph	Minigraph-Cactus	PGGB
Nodes Count	918,744	56,279,782	62,708,282
Edges/Links Count	1,292,003	76,572,277	87,025,931
Segments/Sequence Count	307,012	920,868	62,708,282
Total Length (bp)	1,369,702,218	1,577,591,934	2,931,709,605
Run Time (hh: mm: ss)^b^	6:39:59	7:29:44	38:56:23
Total variants count^c^	163,292	10,049,082	13,839,006
SNPs	0	6,535,280	9,682,418
SVs and INDELs	163,292	4,098,991	4,156,588

^a^SNPs-Single Nucleotide Polymorphisms, INDELs (Insertions and Deletions with size < 50 bp), SVs- Structural Variants with size > 50 bp

^b^Run Time is based on 750 GB RAM and 50 CPUs requested memory on server for all three pipelines

^c^The total variant counts for Minigraph only includes SVs, Minigraph-cactus and PGGB includes all types of variants (SNPs, INDELS, and SVs)

The Minigraph-Cactus and PGGB produce more elaborate graphs compared to minigraph with more nodes and edges/links (Table 6). Similar relative levels of complexities across these different methods were reported by Andreace et al. [235] who developed the human graph pangenomes. In the current analyses, addition of just three assemblies increased the length of the assembly 1.5 to 3 times more compared to a linear reference genome. But, compared to linear pangenomes, graph-based pangenomes identified fewer SNPs and SVs. All three cultivars used in these analyses have been bred for the same geographical region, which suggests that these cultivars are tailored to the environmental conditions, climate, soil type, and other factors specific to that region. Diversity analysis of global tetraploid alfalfa samples, based on 44,757 SNPs, revealed that Chinese genotypes are different from those found elsewhere on the globe [273]. We anticipate if more cultivars from different geographical origin are added in pangenome analyses, we will identify more variants, and more complex graphs will be produced. Moreover, as suggested by Hu et al. [274], the ideal parameters, especially for PGGB, differ among species and typically require several computational trials to properly optimize. As the complexity in the graphs increases it poses a challenge for downstream analyses including alignments, extracting a region of interest from the graph etc., examples of which are shown in the next section.

#### Interpretation of genetic variation and visualization of pangenome graphs

**Fig. 7:**
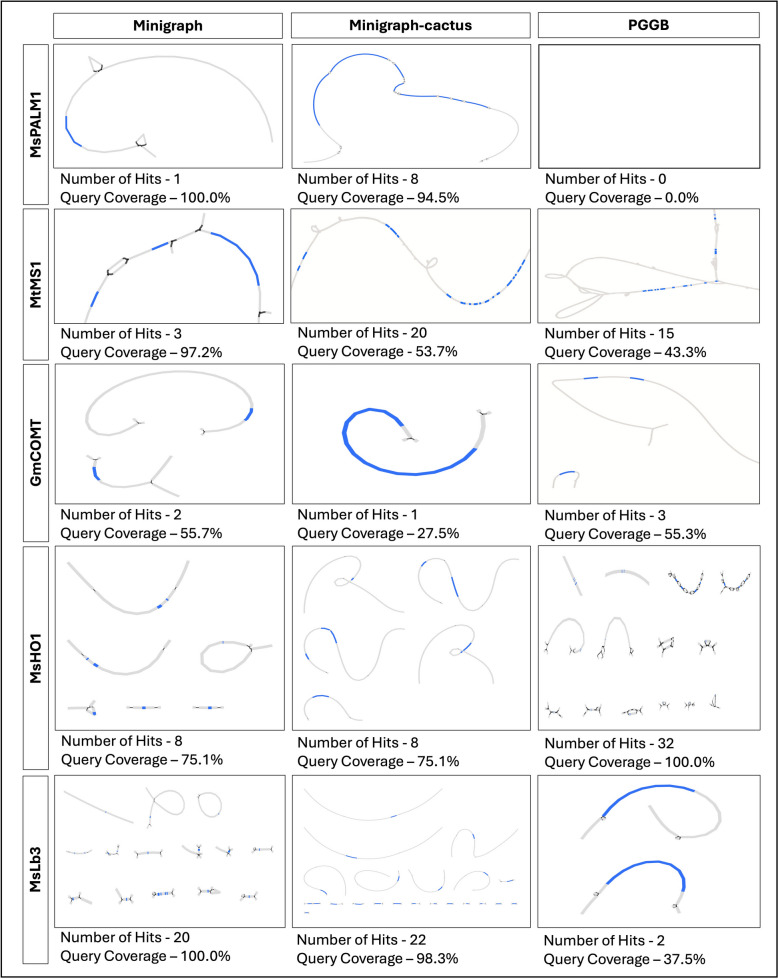
Alignment of five different genes against graph-based pangenomes in alfalfa using BLAST. The blue highlighted regions show the aligned regions on different nodes in the graphs. The five different genes include *Medicago sativa* palmate-like pentafoliata 1 (*MsPALM1*; GenBank accession: HM038483.1), *M. truncatula* PHD finger protein male sterility 1 (*MtMS1*; GenBank accession: XM_003613725.3), *Glycine max* caffeic acid 3-O-methyltransferase (*GmCOMT*; KEGG gene database reference number: gmx:100780100), *M. sativa* chromoplast heme oxygenase 1 (*MsHO1*; GenBank accession: HM212768.1), and *M. sativa* leghemoglobin 3 (*MsLb3*; GenBank accession: M91077.1). These figures were generated with BANDAGE (v. 0.8.1) software using an in-built BLAST feature with default parameters

Multiple software programs have been developed to visualize the pangenome graphs and investigate the different haplotypes and variations in different regions of the genome. These software programs include BANDAGE [212], ODGI [216], Sequence Tube Map [275], among others (Table 4). The first challenge we encountered was the substantial size of the pangenome graphs, particularly Minigraph-Cactus- and PGGB-based graphs, which required significant CPU time and memory just to load these into BANDAGE. To address this, we utilized the Linux version of BANDAGE and executed it on a high-performance graphics processing units (GPU) server with higher memory (32 cores) and processing speed. Then we selected five different gene sequences: *M. sativa* palmate-like pentafoliata 1 (*MsPALM1*) gene (GenBank accession HM038483.1; 765 bp); *M. truncatula* PHD finger protein male sterility 1 (*MtMS1*) (XM_003613725.3; 2,160 bp); *M. sativa* (cultivar Iroquois) leghemoglobin (*MsLb3*) gene (M91077.1; 584 bp); *Glycine max* caffeic acid 3-O-methyltransferase (*GmCOMT*) gene (Kyoto Encyclopedia of Genes and Genomes (KEGG) Genes database reference number gmx:100780100; 1098 bp); and, *M. sativa* chromoplast heme oxygenase 1 (*MsHO1*) gene (GenBank HM212768.1; 852 bp). We wanted to select multiple low to high complexity regions to interpret the variation captured by three graph pangenomes developed in the current study. *MsPALM1* regulates multifoliate leaf formation in alfalfa [35]. *MtMS1* in *M. truncatula* is the ortholog of *Arabidopsis MALE STERILITY1* (*AtMS1*) gene, which encodes a PHD-finger transcription factor and regulates pollen and tapetum development [276]. *MsHO1* plays a crucial role in oxidative responses in alfalfa, contributing to its development and stress regulation [277]. *COMT* is an essential gene responsible for regulating lignin synthesis in plant cell walls [278]. Leghemoglobin (*Lb*) is a heme-containing protein that facilitates oxygen transport in the root nodules of soybean, alfalfa, and other nitrogen-fixing plants [279]. *MsLb3* encodes for the one of the five components of *Lb* protein [280]. *MsPALM1*, *MtMS1*, and *MsHO1* are likely single-copy genes, whereas *GmCOMT* and *MsLb3* are expected to be multicopy genes. *MtMS1* and *GmCOMT* were selected from other legume species to identify their orthologs in alfalfa pangenomes and compare the findings with the linear reference genome. We BLASTed these genes against the three pangenome graphs generated above using BLAST + v2.13.0 software coupled with the BANDAGE using default parameters. The results are presented in Fig. 7. We used the ‘Around BLAST hits’ draw graph option with a distance of 2 (i.e. 2 nodes upstream and downstream of the locus) in BANDAGE to view the BLAST results for all genes. We compared the results with the ZhongmuNo.1 linear reference genome.

The total query coverage for the ZhongmuNo.1 linear reference genome was 100.0%, 97.2%, 55.7%, 75.1%, and 100.0%, with alignment hit counts of 1, 3, 2, 8, and 17 for *MsPALM1*, *MtMS1*, *GmCOMT*, *MsHO1*, and *MsLb3*, respectively. Minigraph showed identical coverage to the linear genome in BLAST results, but the number of alignment hits for the *MsHO1* and *MsLb3* genes was higher (Fig. 7, column 1), as the alignment was split on a node for both genes. In Minigraph-cactus, total query coverage decreased for four of the five genes, with alignments frequently split across a single node, resulting in a higher number of hits compared to the linear genome (Fig. 7, column 2). This tool captured multiple SNPs and a range of insertions, from small to large, in these loci, represented by various nodes and edges. In PGGB, no alignment was found for the *MsPALM1* gene, as none of the graph segments/sequences aligned to it. Query coverage for all genes was significantly reduced in the PGGB pangenome graph, except for the *MsHO1* gene, where coverage increased to 100% compared to 75.1% in the linear genome, although the alignment was split (Fig. 7, column 3). These findings are very similar to those reported by Andreace et al. [235] for human pangenomes, particularly for the visualization and interpretability of low- and high-complexity regions in 2–10 haplotypes based pangenome graphs. The key difference was that, even with just three genomes, graph pangenomes in alfalfa are already highly complex, particularly with PGGB, compared to human genomes. This discrepancy is likely due to their use of gfatools to compress the nodes in Minigraph-Cactus and PGGB, and the complexity of plant genomes over human genomes. Their method of extracting the regions (subgraphs) was also different and more complicated than our straightforward approach. These tools differ notably in how they represent genetic variation. Overall, as graph complexity increases, it becomes more challenging to extract regions of interest, underscoring the complexity of plant genomes. This suggests that constructing a locus-based pangenome could be a promising approach in highly heterozygous polyploids.

This also illustrates the challenge that local alignment tools encounter when aligning a sequence within a graph-based pangenome, especially when complexity increases. Visualizing linear pangenomes is significantly simpler compared to graph-based pangenomes. Web-based browsers such as GBrowser [281], JBrowse 2 [282], Integrative Genomics Viewer (IGV) [283], and PANACHE- the PANgenome Analyzer with CHromosomal Exploration [214] offer convenient, user-friendly formats for exploring gene- and sequence-based pangenomes. PPanG [284] provides a unique opportunity to visualize graph-based pangenomes directly in a web browser. It leverages the sequence-tube-map viewer and includes a linear view for each individual genome using JBrowse2. Integrating graph-based pangenomes into linear pangenome web-browser is a good start. Developing better user-friendly tools is important as they play a critical role in simplifying complex pangenomes, making it more accessible to researchers across various fields.

For conducting genome-wide studies, using large, elaborate, and complex graphs produced by Minigraph-cactus, PGGB, and Cuttlefish may be more appropriate as they capture all kinds of variation between genomes as demonstrated by Liao et al. [236], Cleary et al. [234], Manuweera et al. [285], and Zhou et al. [230]. In nearly all published studies on plant pangenome graphs to date, minigraph or VG Toolkit has been the preferred choice, likely due to the greater complexity of plant genomes compared to those of animals. Minigraph is the appropriate option when analyzing a large number of genomes, as the complexity significantly escalates with an increase in the number of genomes. Moreover, this tool has the shortest runtime (Table 6).

## Conclusion

Although advancement in sequencing technology has brought down the cost of genome sequencing for complex plant genomes, highly repetitive regions of the genome remain a challenge to assemble. Nevertheless, comparative genomic analyses provide valuable insights into crop genomes. Pangenome resources in different crops are being assembled and are proving useful to the plant biology community by aiding in comprehending the significance of core and dispensable genes in diverse biological functions [67, 70, 86], identifying novel genes in the reference genomes [61], conducting more precise allele specific gene editing [286], investigating genome-wide patterns of genetic variations and introgressions [106, 108, 110, 257], and finding associations between SVs and various traits [85, 101, 287]. After developing such resource, it is important to make it accessible to the scientific community in an easy-to-understand and use format. The size of a pangenome is shaped by factors such as reproductive mode, ploidy level, and the genetic diversity sampled within a species. There is a pressing need for the development of more advanced tools such as PPanG [284] to effectively visualize the vast amount of genetic variation captured by graph-based pangenomes, particularly in cross-pollinated, highly heterozygous species.

Many crops online portals e.g., RPAN [288] and RiceSuperPIRdb [289] in rice, MaizeGDB [290] and ZEAMAP [291] in maize, The Wheat Panache Web Portal [292] and Wheat Pangenome [66] in wheat, PepperPan [87] in peppers, and PanSoy [86] in soybean, have been developed allowing plant biologists and the breeding community access and visualization in an easy-to-read format. Accurately assembling a plant pangenome requires expertise in bioinformatics, genetics, and related fields. Graph-based pangenome development is a promising technique but is still in its infancy. The array of available formats and incompatibility of these formats with analysis tools presents challenges to wide adoption of graph-based approaches. There are multiple tools to choose from and each has its pros and cons. Researchers must develop a suitable pipeline which works for a particular crop by combining these multiple tools. The resources listed here will provide researchers a stepwise guide to develop a pangenome based on the available resources in each crop.

## Data Availability

No datasets were generated or analysed during the current study.

## Abbreviations

CADL: Cultivated Alfalfa at Diploid Level
CNVs: Copy Number Variations
FRs: Frequent Regions
GFA: Graphical Fragment Assembly
GFF: General Feature Format
Hi-C: High Throughput Chromosome Conformation Capture
INDELs: Insertions and Deletions
PAVs: Presence or Absence Variations
PGGB: PanGenome Graph Builder
rGFA: reference GFA
SNPs: Single Nucleotide Polymorphism
SVs: Structural Variants
TEs: Transposable Elements
VCF: Variant Calling Format
VG: Variation Graph
